# Functional minigenome system reveals polymerase features of swine orthopneumovirus

**DOI:** 10.1128/jvi.00363-26

**Published:** 2026-05-19

**Authors:** Charles-Adrien Richard, Manuel Feliciano, Mathilde Omond, Christine Krempl, Jean-François Eléouët

**Affiliations:** 1 Unité de Virologie et Immunologie Moléculaires (VIM), UMR0892, INRAE, UVSQ, Université Paris-Saclay 27048, Jouy-en-Josas, France; 2 Institute for Virology and Immunobiology, Julius-Maximilians-Universität 9190 , Würzburg, Germany; University of Freiburg, Freiburg, Germany

**Keywords:** swine orthopneumovirus, SOV, pneumovirus, minigenome, RNA polymerase, cellular protein phosphatase 1, PP1, reverse genetics

## Abstract

Swine orthopneumovirus (SOV), a recently identified porcine pneumovirus, has been detected in pig farms worldwide; however, its pathogenicity and molecular biology remain poorly understood. To facilitate the study of SOV replication and transcription, we developed a functional minigenome system based on consensus sequences from multiple strains of SOV and related pneumoviruses. Here, we constructed and optimized this system in BSRT7/5 cells, revealing that the RNA-dependent RNA polymerase (RdRp) activity depends on a conserved protein phosphatase 1 (PP1) binding site within the phosphoprotein P, as a single F131A substitution markedly reduced polymerase function. Additionally, we identified and characterized the M2-1 binding site on P, which is essential for viral transcription. These findings provide new insights into SOV polymerase complex requirements and establish a foundation for reverse genetics approaches to rescue infectious viruses, advancing our understanding of SOV biology and its potential role in porcine respiratory disease.

**IMPORTANCE:**

Recently, a newly identified porcine pneumovirus, swine orthopneumovirus (SOV), was detected in pig farms in different countries. Although detected mainly in sick animals, this virus has not been isolated yet and its pathogenicity remains to be determined. We started by setting up a minigenome system with a view to develop reverse genetics and rescue infectious virions. This minigenome system was used to study the functioning of the SOV RNA polymerase and compared it with RSV. Although some similarities exist between SOV and RSV, the RdRp of RSV cannot rescue the SOV minigenome. SOV seems to belong to another genus/genogroup of pneumoviruses, which includes PVM and the canine pneumovirus. Our functional minigenome paves the way for reverse genetics of SOV and determination of its pathogenicity in different host species.

## INTRODUCTION

The first suspicion of the presence of pneumoviruses in pigs was initiated by a serological study conducted in Northern Ireland in 1998, in which the presence of antibodies cross-reacting against bovine respiratory syncytial virus-infected cells was observed (1). A study conducted in the USA in 2016 confirmed the existence of a pneumovirus in pigs using metagenomic sequencing (2). Sequence analysis of this virus, which was named swine orthopneumovirus (SOV), revealed that it is closely related to pneumonia virus of mice (PVM) and canine pneumovirus (CPV), but clearly divergent from human (HRSV) and bovine (BRSV) respiratory syncytial orthopneumoviruses and metapneumoviruses, suggesting that SOV, PVM, and CPV could form a third subfamily in the *Pneumoviridae* family (2, 3). The published SOV genomic sequence was further used by other groups from other countries, and SOV was detected in France in 2018 (4), in Spain in 2022 (5), in Germany (6) and Korea (3) in 2023, and finally in Sweden in 2025 (7). Most of these studies suggested that SOV was present in young pigs with respiratory diseases but most frequently in combination with other respiratory viruses (3, 5, 6) or bacteria (7). However, to date, no one has been able to isolate this virus from animals. Thus, the role of SOV in the pathogenesis of porcine respiratory disease complex (PRDC) is uncertain and requires further study. To clarify this issue, we aimed to develop a reverse genetics system for rescueing infectious SOV particles. We started this work based on the sequences published by Park et al. (3) with the aim of engineering an SOV minigenome system, the first step to develop reverse genetics. Sequence comparison between SOV and HRSV revealed the presence of putative binding sites for the SOV M2-1 protein and the cellular protein phosphatase 1 in the SOV phosphoprotein P. Using a bicistronic minigenome, M2-1 was found to be essential for the transcription of both genes. The role of PP1 in the SOV RdRp activity was confirmed by using site-directed mutagenesis of the putative PP1 binding site.

## RESULTS

### Development of an SOV minigenome system

**Fig 1:**
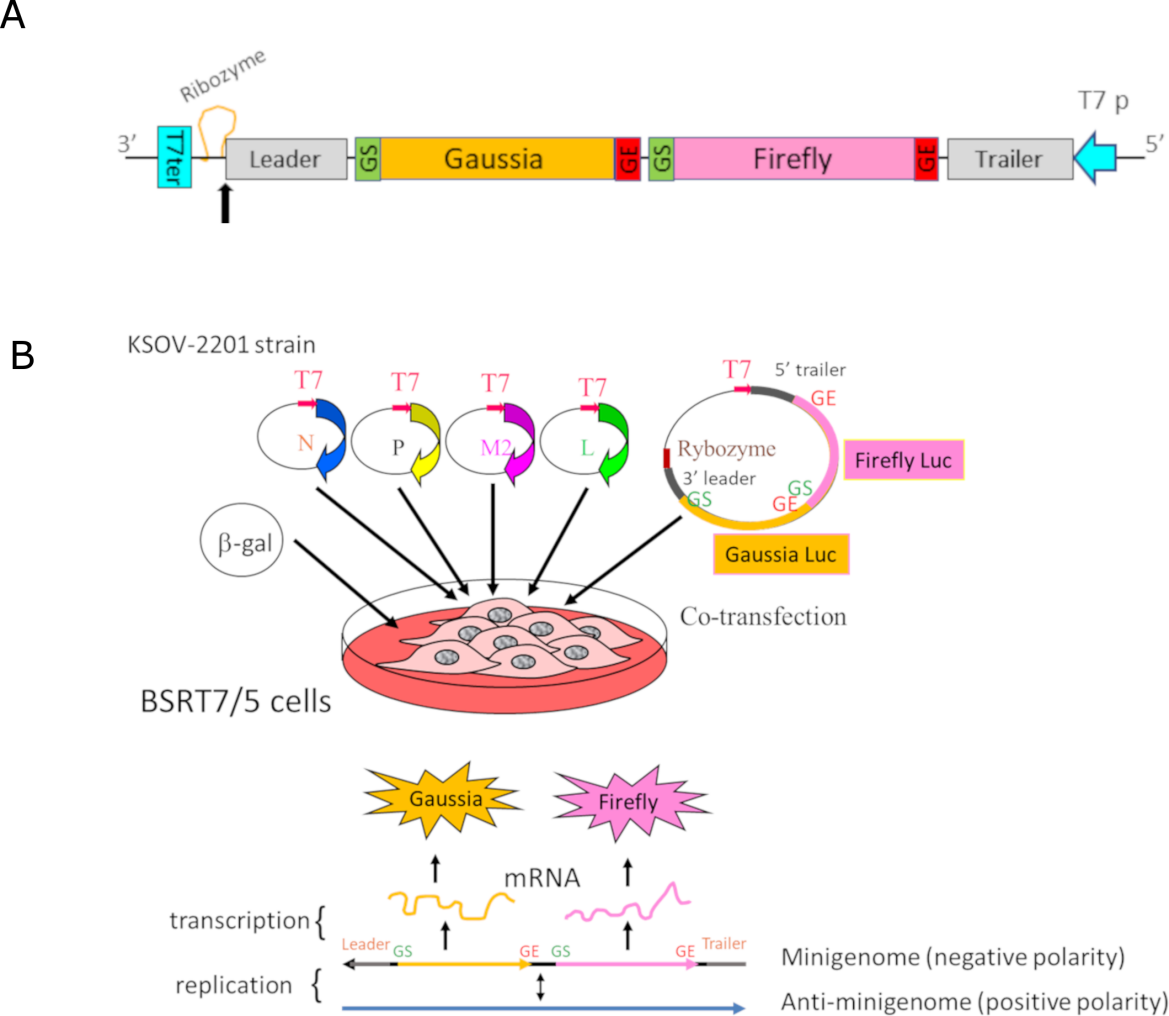
(**A**) Schematic representation of the elements constituting the SOV minigenome sequence cloned into pUC57 vector. The black arrow indicates the cleavage site induced by the hepatitis delta virus (HDV) ribozyme (yellow). The full sequence is framed by a T7 promoter (T7pr, blue arrow) and a T7 terminator (T7ter, blue box) sequences. The Leader (NewLeader2) and the Trailer sequences are shown as gray boxes; gene start (GS, green) and gene end (GE, red) signals are derived from KSOV2201. The Gaussia and Firefly and luciferases genes are shown as orange and magenta boxes, respectively. (**B**) Schematic representation of the minigenome system assay. Plasmids expressing the N, P, M2-1, and L proteins of KSOV-2201 under the control of a T7 promoter were co-transfected with the minigenome-encoding plasmid together with a β-Gal reporter vector for transfection standardization. Twenty-four hours after transfection, the cells were lysed and luciferase signals were quantified.

**Fig 2:**
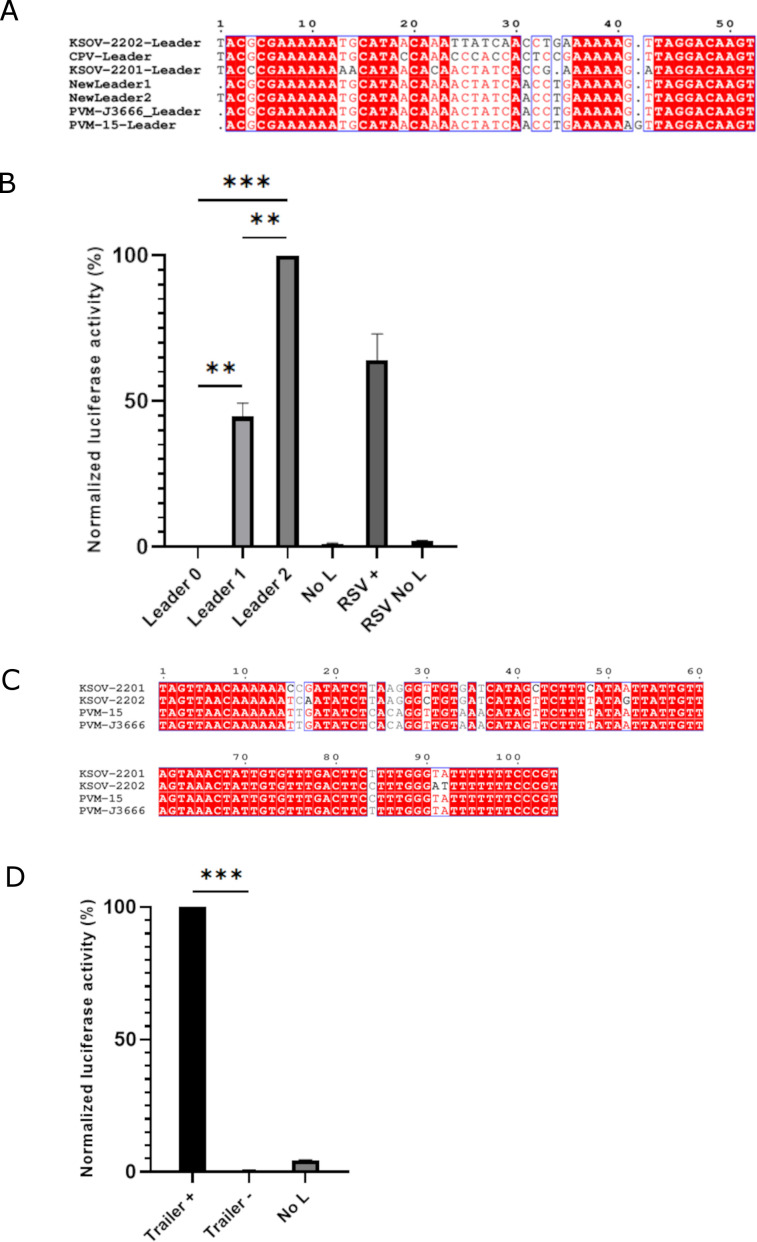
Evaluation of the functionality of SOV Leader and Trailer sequences using the SOV minigenome. (**A**) Sequence alignments between the two published SOV Leader cDNA sequences KSOV-2201 (GenBank accession number: OR701947.1) and KSOV-2202 (GenBank accession number: OR701948.1), and those of one CPV, strain Bari/100-12/ITA/2012 (GenBank accession number: KF015281.1), and two PVM strains, J3666 (GenBank accession number: AY743909.1) and 15 (GenBank accession number: AY729016.1). The two newly synthesized SOV leader sequences are also shown. (**B**) Comparison of SOV RNA polymerase activity between the three constructs. BSRT7/5 cells were transfected with plasmids encoding the SOV P, N, L, and M2-1 proteins and the different pMiniG, together with pCMV-βGal for transfection standardization. Viral RNA synthesis was quantified by measuring the firefly luciferase activity after cell lysis 24 h after transfection. Each luciferase minigenome activity value was normalized based on β-galactosidase expression and was the average of experiments performed in triplicate. An HRSV minigenome (13) with (RSV+) or without (RSV NoL) the pL plasmid was used as a control. (**C**) Sequence alignments between the two published Korean SOV Trailer sequences and two PVM strains. (**D**) SOV minigenome assay using the pMiniG2 with (Trailer+) or deleted (Trailer−) of the trailer sequence performed as in panel B. A negative control was performed by removing the pL plasmid from the transfection mixture. Error bars represent standard deviations. **P < 0.01, ***P < 0.001 (unpaired *t*-test with Welch’s correction).

Our long-term goal is to rescue SOV infectious particles and to determine their pathogenicity. Therefore, we started by setting up a minigenome system with a view to develop reverse genetics and rescue recombinant viruses. A minigenome system was constructed based on the sequences published by Park et al. (3). As a start, we used the sequence of the strain KSOV-2201. A minigenome containing a T7 promoter, 5′ Trailer, 3′ Leader sequences, gene start (GS) and gene end (GE) sequences of this clone as well as genes for Firefly and Gaussia luciferases and a ribozyme at the 3′ end was synthesized and cloned into pUC57 (see Fig. 1; Fig. S1). In parallel, the genes encoding for SOV L, N, P, and M2-1 proteins, which are expected to constitute the RNA-dependent RNA polymerase (RdRp) complex, were also synthesized and cloned into the pCite vector under the control of a T7 promoter. These five plasmids were co-transfected into BSRT7 cells that constitutively express the T7 RNA polymerase. An HRSV minigenome previously developed in our laboratory (8) was used as a control. The ratio between plasmids encoding the different genes and the minigenome was based on those previously used for PVM reverse genetics (9–12). If functional, the luciferase activity should reflect the SOV polymerase activity. Unfortunately, no luciferase signal was obtained with the first minigenome construct containing the KSOV-2201 published sequences. We, therefore, assumed that incorrect sequences in our constructs were responsible for the non-functioning of this minigenome. Thus, we aligned sequences from the different SOV strains available in GenBank together with sequences of PVM strains 15 and J3666, and CPV strain Bari/100-12/ITA/2012, and some differences were evident between the different viruses and strains (Fig. 2; Fig. S2). As a first step, we modified the leader sequence based on sequence alignments (Fig. 2A). More specifically, nucleotide differences between KSOV-2201 and the other viruses were found at positions 1, 4, 13, 14, and 19. Two different sequences were synthesized, one based on the consensus sequence (Leader1), and the other one (Leader2) with the same sequence than Leader1 but containing a 5′ supplementary T on the cDNA sequence (Fig. 2A) and introduced into the SOV minigenome. As shown in Fig. 2B, the SOV minigenome with the Leader2 sequence was functional and had a higher activity than the Leader1 construct, and the original sequence (Leader0) gave no signal at all. We, thus, used this clone (plasmid pMiniG2) for subsequent experiments.

We then aligned the trailer sequences of the two KSOV strains with those of the two PVM strains (Fig. 2C), but no significant changes were observed in the KSOV-2201 sequence was observed. Nevertheless, we deleted the trailer region from MiniG2 to test its functionality. As shown in Fig. 2D, the deletion of the trailer sequence abolished both Gaussia and firefly luciferase activities, indicating that genome encapsidation and replication were abolished, resulting in loss of transcription of the reporter genes. These results indicate that the trailer sequence present in the minigenome is functional.

**Fig 3:**
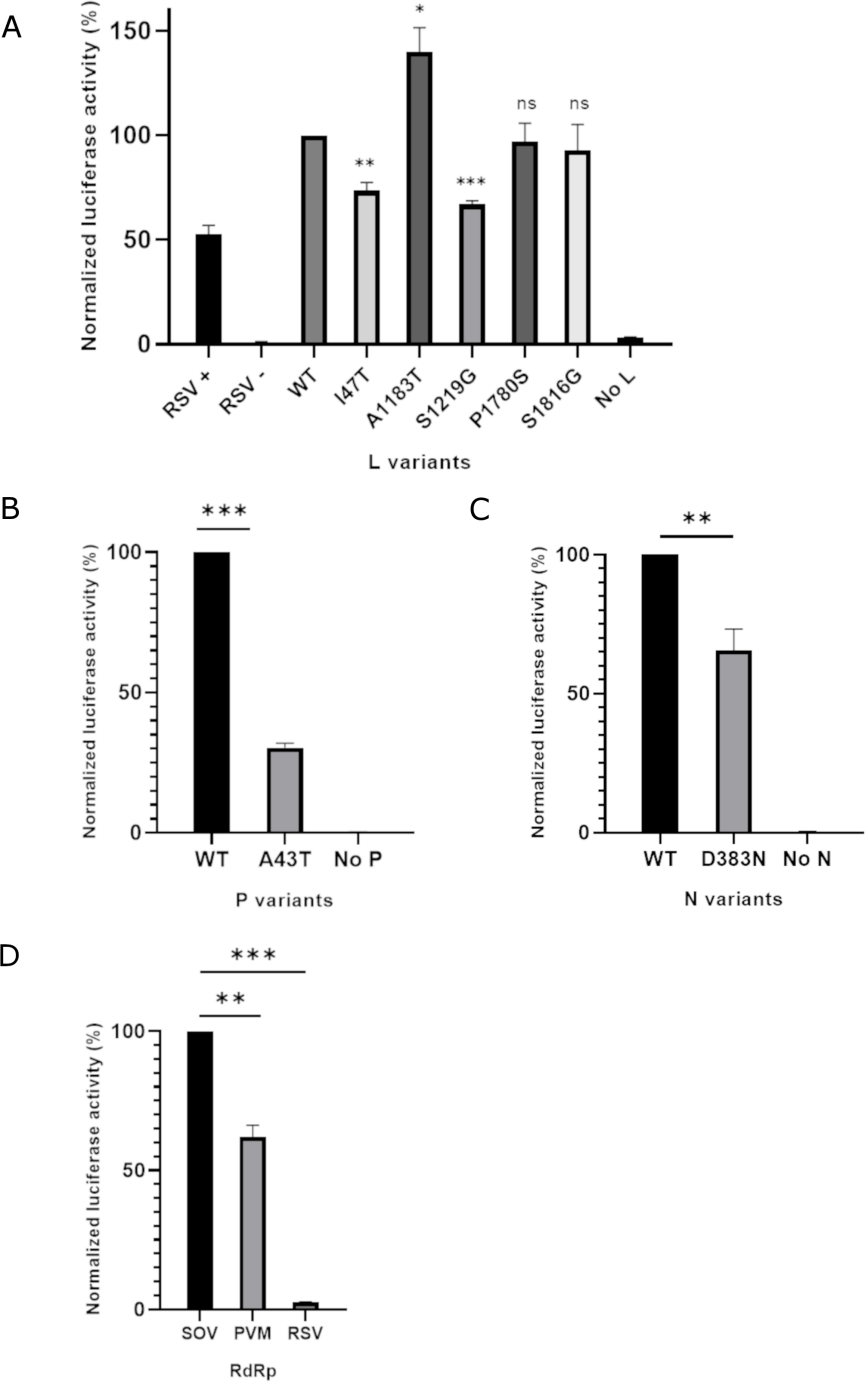
Effect of L, P, and N variants on the SOV polymerase activity. BSRT7/5 cells were transfected with plasmids encoding the WT or variant SOV L (**A**), P (**B**), and N (**C**) together with pM2-1, the pMiniG2 and pCMV β-gal for transfection standardization. Amino acid substitutions were based on sequence variations from the consensus sequence identified for each gene, as shown in Fig. S2. (**D**) Complementation assays with SOV, PVM, and RSV RdRp and the SOV minigenome (pMiniG2). A previously established RSV minigenome system (13) was used as a control. Luciferase activity, reflecting viral RNA synthesis, was measured 24 h after transfection, normalized to the β-galactosidase activity, and expressed as a percentage of the WT protein activity. The mean value ± SD from three independent experiments performed are shown. ns, non significant, *P < 0.05, **P < 0.01, ***P < 0.001 (unpaired *t*-test with Welch’s correction).

In the next step, we aligned the sequences of the KSOV2201 L, N, P, and M2-1 genes that were cloned in our expression vectors with those of SOV-57, KSOV-2201, KSOV-2202 strains and the three other viruses PVM and CPV described above to search for some potential mutations that could affect the RdRp activity. These sequence alignments revealed five potential amino-acids in L (I47, S73, A1183, S1219, and P1780), one in M2-1 (R174), one in N (D383), and one in P (A43) genes, as indicated by arrows in Fig. S2. Different amino acids were substituted for the consensus amino acids and tested with the minigenome except for M2-1, which has a lysine instead of an arginine at the C-terminus. The results are shown in Fig. 3. None of the substitutions showed a significant positive effect on the RNA polymerase activity for N and P. However, for L, one substitution (A1183T) showed a 40% increase in the Luc reporter activity. This residue should be considered in the future development of reverse genetics and virus rescue.

### The SOV minigenome can be rescued by PVM but not by the HRSV RdRp complex

Although SOV has been classified in the genus *Orthopneumovirus* (2), sequence comparisons between SOV and the other members of the *Pneumoviridae* family have shown that SOV, PVM, and CPV are closely related and clearly diverge from both other orthopneumoviruses and metapneumoviruses, including cis-acting elements that are recognized by the RdRp machinery, and in particular Gene Start sequences (2, 3). To test whether the cis-acting elements of SOV could be cross-recognized by HRSV RdRp, the SOV minigenome was cotransfected with either plasmids coding for HRSV or PVM RdRp complexes. As shown in Fig. 3D, PVM, but not HRSV, RdRp could rescue the SOV minigenome, with a ~ 60% activity with PVM RdRp compared to SOV RdRp.

### Role of SOV M2-1 in transcription

**Fig 4:**
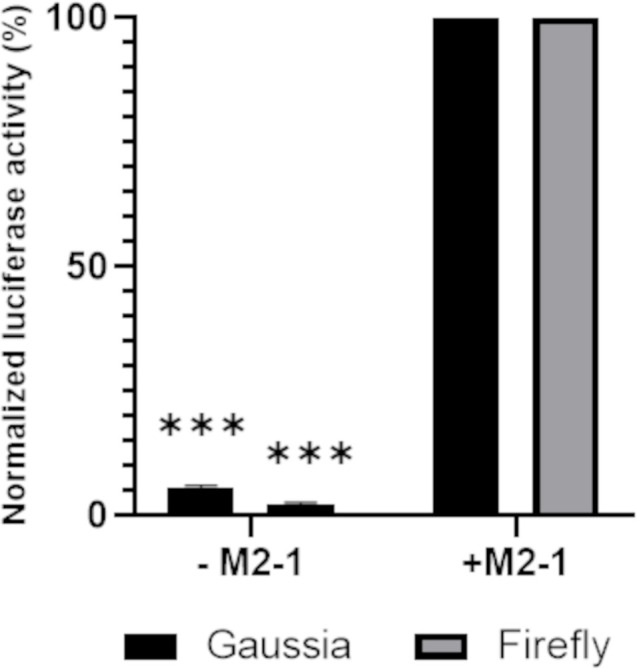
SOV M2-1 is required for efficient viral transcription. BSRT7/5 cells were transfected with SOV pP, pN, pL, and pM2-1 (+M2-1) or empty pCite (−M2-1) plasmids and the pMiniG2 expressing the SOV minigenome containing Gaussia and firefly luciferase reporter genes, together with p-β-Gal constitutively expressing β-galactosidase. Luciferase activities, measured 24 h after transfection, were normalized to β-galactosidase activity, and the luciferase activity gained with M2-1 was set to 100%. The mean values and confidence intervals (error bars) were obtained from three separate experiments performed in duplicate. ***P < 0.001 (unpaired *t*-test with Welch’s correction).

Similar to other pneumoviruses, the SOV M2 gene encodes for two potential ORFs, M2-1 and M2-2. For HRSV, it was determined that M2-1 serves as an elongation (or processivity) transcription factor to enable the synthesis of full-length mRNAs (14). Further experiments using a bicistronic minigenome showed that M2-1 is essential for efficient transcription of the second and downstream genes, transcription of the first gene being reduced, but not abrogated, in the absence of M2-1 (14, 15). These studies indicated that the major role of M2-1 during transcription is to facilitate intragenic elongation as the polymerase transcribes RNA from the gene start to gene end signals (16). In contrast, using a PVM monocistronic reporter minigenome, Dibben et al. observed that M2-1 is essential for PVM RNA synthesis of the 3′ end reporter gene under the control of the leader region (11). We revisited this question for SOV using the functional bicistronic minigenome. As show in Fig. 4, in the absence of SOV M2-1, both Gaussia and firefly luciferase signals were reduced to undetectable levels, indicating that transcription was abrogated, as observed with PVM.

### SOV N and P proteins are sufficient to induce cytoplasmic IB-like structures

**Fig 5:**
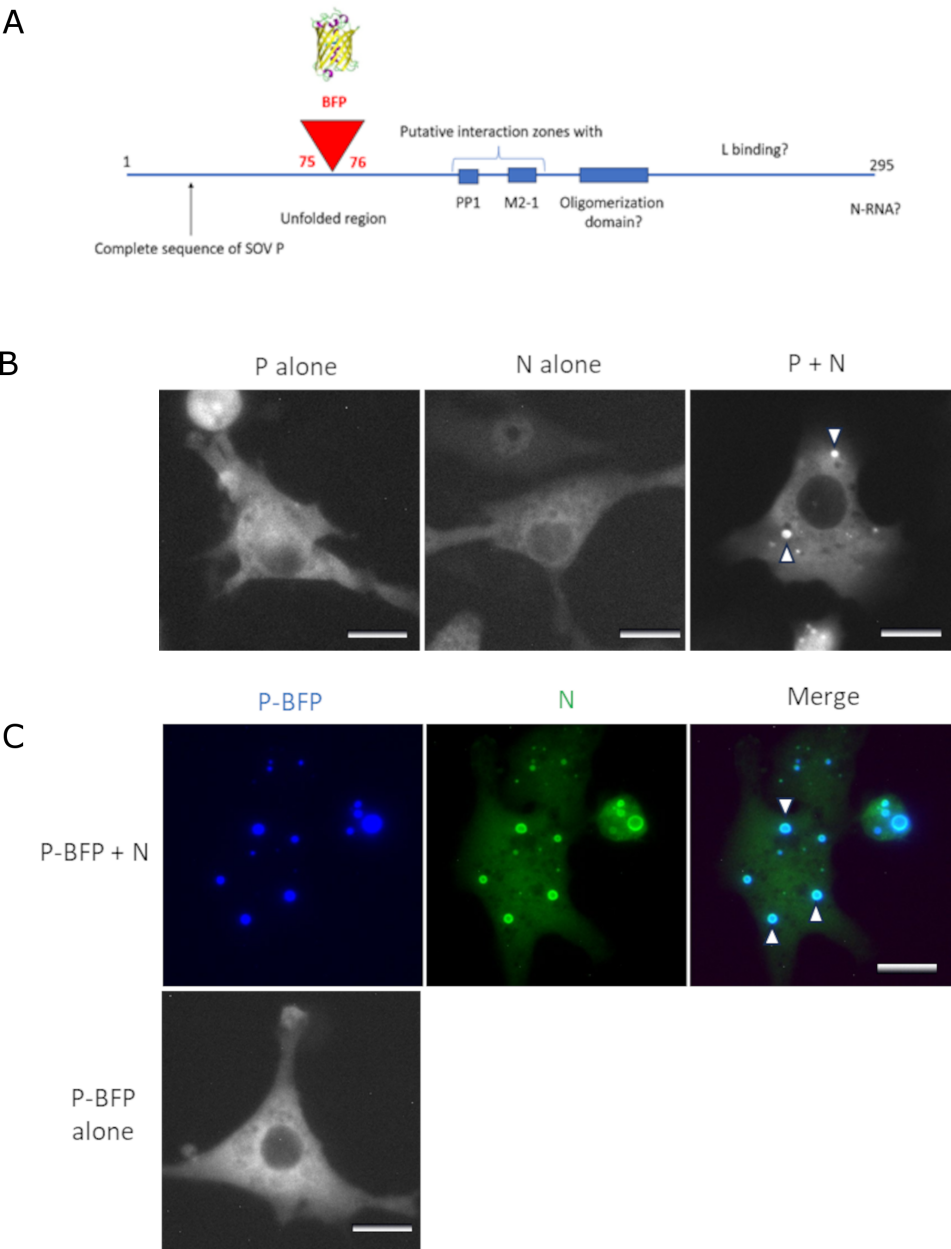
An insertion of BFP in P does not affect the formation of IB-like structures when co-expressed with N. (**A**) Schematic illustration of the insertion of BFP into the N-terminal arm of SOV P. The blue line represents the complete sequence of SOV P, which contains 295 amino acids. The red arrowhead represents the insertion site of BFP in P, between amino acids 75 and 76. The insertion site was located in a predicted unfolded region. The blue boxes represent, from left to right, the putative interaction zones between SOV P and PP1, M2-1, and the potential oligomerization domain of P. The putative interaction domains of P with the L polymerase and RNA-N nucleocapsid are located in the C-terminal region of P. (**B**) SOV P and N form IB-like structures when co-expressed in living cells. BSRT7 cells were transfected with either pP alone, pN alone, or co-transfected with pP + pN (P + N). Cells were fixed and labeled with a rabbit polyclonal anti-N and anti-P sera and detected with a secondary goat anti-rabbit antibody, and colocalization of N with P was analyzed by fluorescence microscopy (**C**) P-BFP formed IB-like structures when co-expressed with N. pP-BFP plasmid was transfected in BSRT7 cells alone or together with pN, cells were fixed, labeled with a rabbit polyclonal anti-N sera and detected with a secondary goat anti-rabbit antibody, and observed by fluorescence microscopy. White arrows indicate IBs. Scale bar, 20 µm.

Pneumoviruses replication and transcription take place in the cytoplasm of infected cells and, for the other pneumoviruses HRSV, BRSV and human metapneumovirus (HMPV), it was shown that these activities take place in cytoplasmic inclusion bodies (IBs), corresponding to viral factories, where RNA synthesis occurs, and that concentrate all the components of the RdRp machinery (17–21). These IBs are formed by liquid-liquid phase separation, and the minimal requirement for their formation is the interaction between RNA, N, and P proteins (17, 18, 22–26), although for HMPV, P was found to be capable of inducing phase separation independently before recruiting N to liquid-like bodies (27). To determine whether IBs are also formed by N + P co-expression in the case of SOV, cells were co-transfected with plasmids pN and pP, fixed, labeled with anti-P and anti-N antibodies, and observed by fluorescence microscopy. As shown in Fig. 5B, the co-expression of SOV N and P proteins was necessary and sufficient to induce the appearance of cytoplasmic inclusion bodies resembling those observed in other pneumovirus infections.

To facilitate the *in cellula* observations of IBs under the microscope, we searched for a place to insert a fluorescent tag, blue fluorescent protein (BFP), in the SOV P protein that should minimally affect its interaction with the N protein. The structure predictions are shown in Fig. S3. Although the N- and C-terminal regions of P were predicted to be disordered, we had to exclude potential regions of interaction with partners that could fold after binding. We excluded the C-terminal region (199–295) since it was identified as an L and N-RNA binding region that folds upon binding with its partners for HRSV (28, 29) (Fig. 5A). The predicted oligomerization domain (residues 178–198) was also excluded, and we focused on the N-terminal region. Two predicted short helices (residues 10–23 and 142–155) were also excluded, as well as the putative PP1 (residues 125–132) and M2-1 (141–155) binding regions (see below), and the 30 most N-terminal residues that were shown to bind to the RNA-free N0 for HRSV (30). Thus, we chose the proline-rich region 61-RVAANLTNPSVPPS**TP**PSIPP-81 for insertion and BFP was inserted between residues T75-P76. As shown in Fig. 5C, fluorescent inclusion body-like structures were observed in cells transfected with N and P-BFP expression plasmids, whereas N, P, and P-BFP showed patterns of diffuse cytoplasmic distribution when expressed alone.

### Identification and functionality study of M2-1 and PP1 binding sites on SOV P

**Fig 6:**
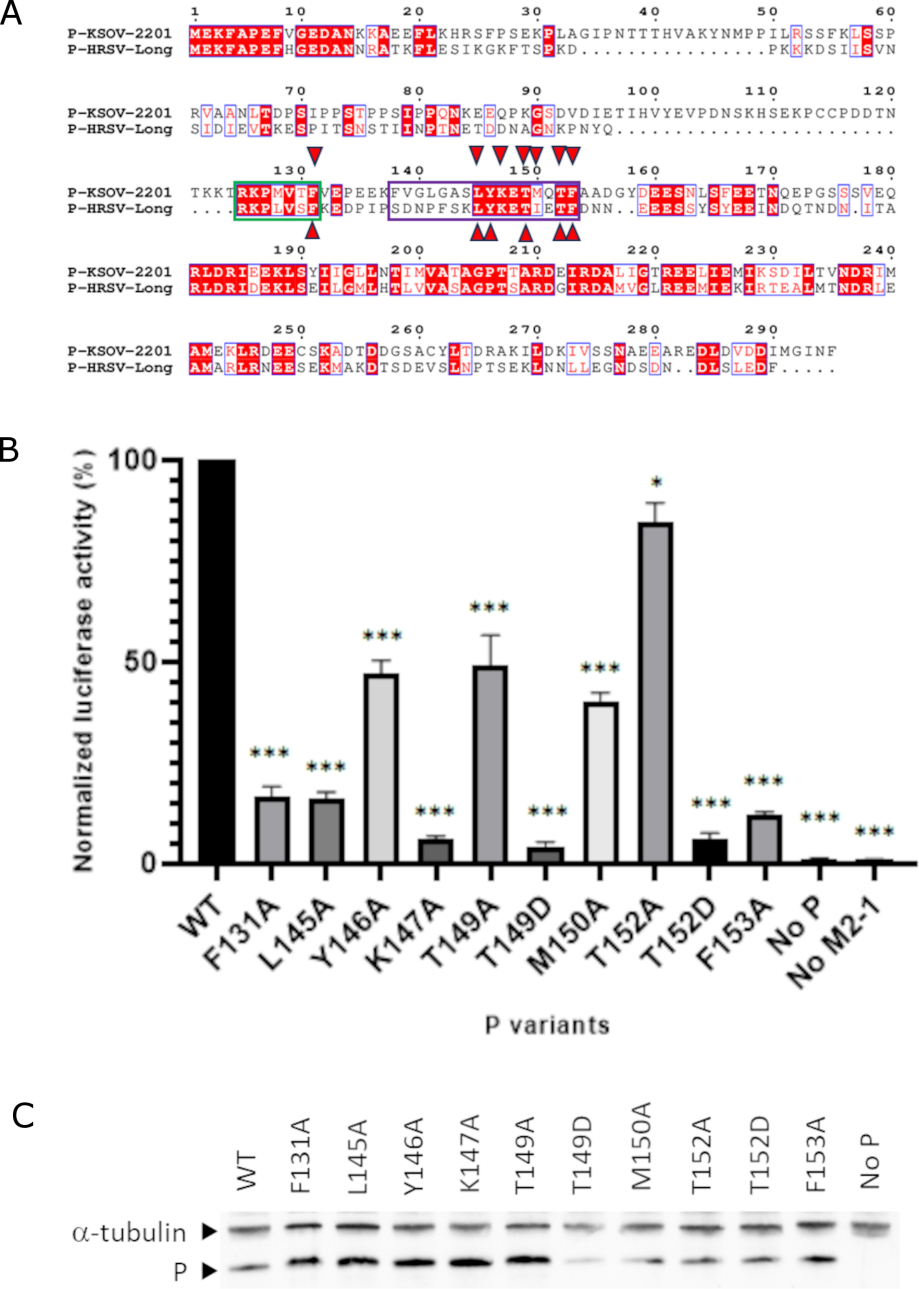
Identification of putative binding sites for PP1 (green frame) or M2-1 (purple frame) on the SOV P protein. (**A**) HRSV (strain Long) and SOV (strain KSOV2201) P proteins were aligned using ClustalW and analyzed using ESPript3. The RSV P interaction sites with PP1 and M2-1 are boxed in green and purple, respectively. Amino acid residues identified on HRSV P as critical for interactions with PP1 and M2-1, and the RdRp activity are indicated by red arrows. The residues identified on the SOV P protein (this study) are also indicated by red arrows. (**B**) Effect of mutations in the M2-1 and PP1 binding sites of SOV P on the minigenome activity. BSRT7 cells were transfected with plasmids encoding the WT N, M2-1, and L proteins, the pMiniG2 and WT or mutated P and analyzed as described in the legend of Fig. 3. (**C**) Western blot showing the expression levels of P variants in the lysates from transfected BSRT7 cells.

Using the SOV minigenome with the Leader2 sequence system, we began to characterize some aspects of the SOV RdRp complex. First, we aimed to determine whether SOV P interacts with M2-1 and the cellular phosphatase PP1, as found for HRSV, recruiting them into IBs for efficient RNA synthesis. M2-1 and the cellular protein phosphatase PP1 binding sites were identified on the HRSV P protein in the N-terminal disordered region (31, 32). Sequence alignment between HRSV and SOV phosphoproteins is shown in Fig. 6A. This revealed that putative binding sites for PP1 and M2-1 are also present on SOV P at residues 125–131 (PP1) and 138–153 (M2-1). To validate both putative sites, some residues identified on HRSV P as critical for the interactions were substituted on their SOV counterpart, in particular residue F131 for PP1 and L145, Y146, K147, T149, M150, T152, and F153 for M2-1 putative binding sites. These residues were substituted with A, and the results obtained with the minigenome assays are shown in Fig. 6B. The single substitutions F131A (PP1 site), L145A, K147A, and F153A (M2-1 site) all strongly affected the SOV polymerase activity as determined by measuring the firefly luciferase activity using the minigenome system, in agreement with our hypothesis. For T149 and T152, only a T→D substitution strongly reduced the RdRp activity, suggesting that phosphorylation of these residues could modulate the M2-1 binding to P. In Fig. 6C, Western blots show that the drop in transcription efficiency was not due to a defect in the expression of the P variants even though the expression of some variants, such as 149D, was reduced.

**Fig 7:**
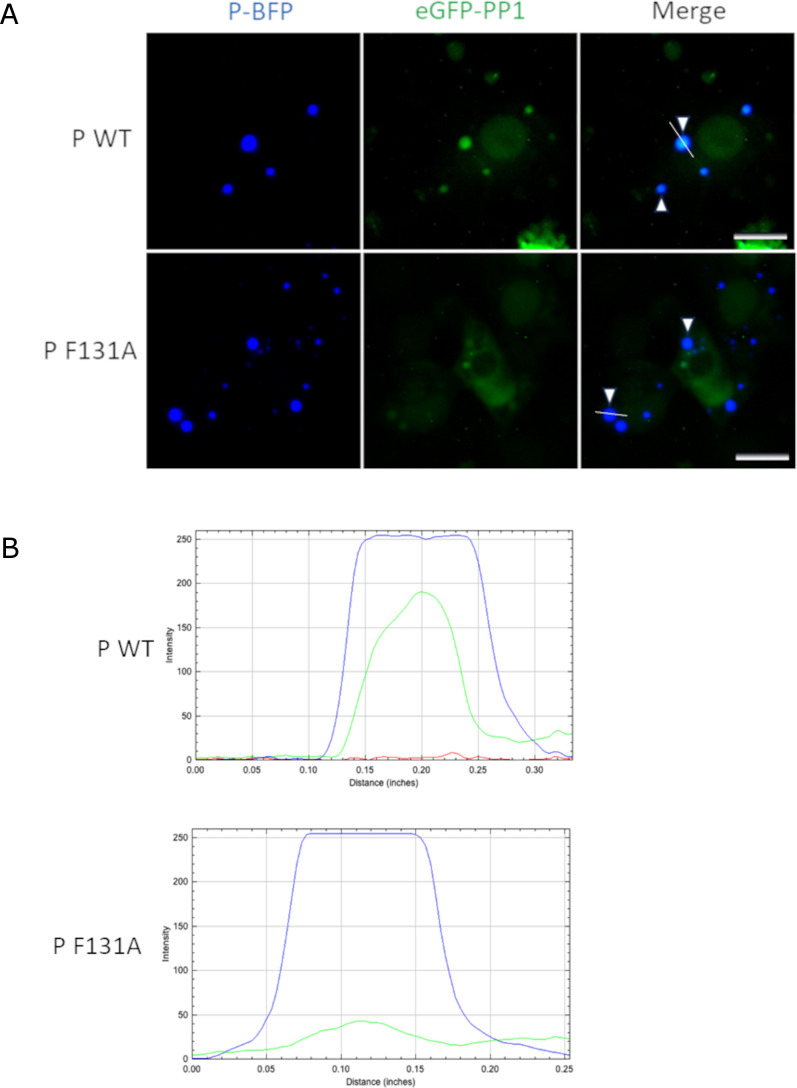
Effect of a point mutation in the PP1 binding site of P on protein localization in living cells. (**A**) BSRT7 cells were transfected with plasmids encoding the N, P-BFP (WT or F131A variant), and eGFP-PP1 proteins as indicated. Twenty-four hours post-transfection, cells were fixed and labeled with a rabbit polyclonal anti-N antibody and detected with a secondary goat anti-rabbit antibody. The colocalization of proteins in IBs was analyzed using fluorescence microscopy. Scale bars, 20 µm. (**B**) Blue and green fluorescence, corresponding to P and EGFP-PP1, respectively, was quantified using ImageJ software from white bars in pictures shown in panel A. When PF131A was used instead of P WT, the green signal in the section was reduced to background level.

**Fig 8:**
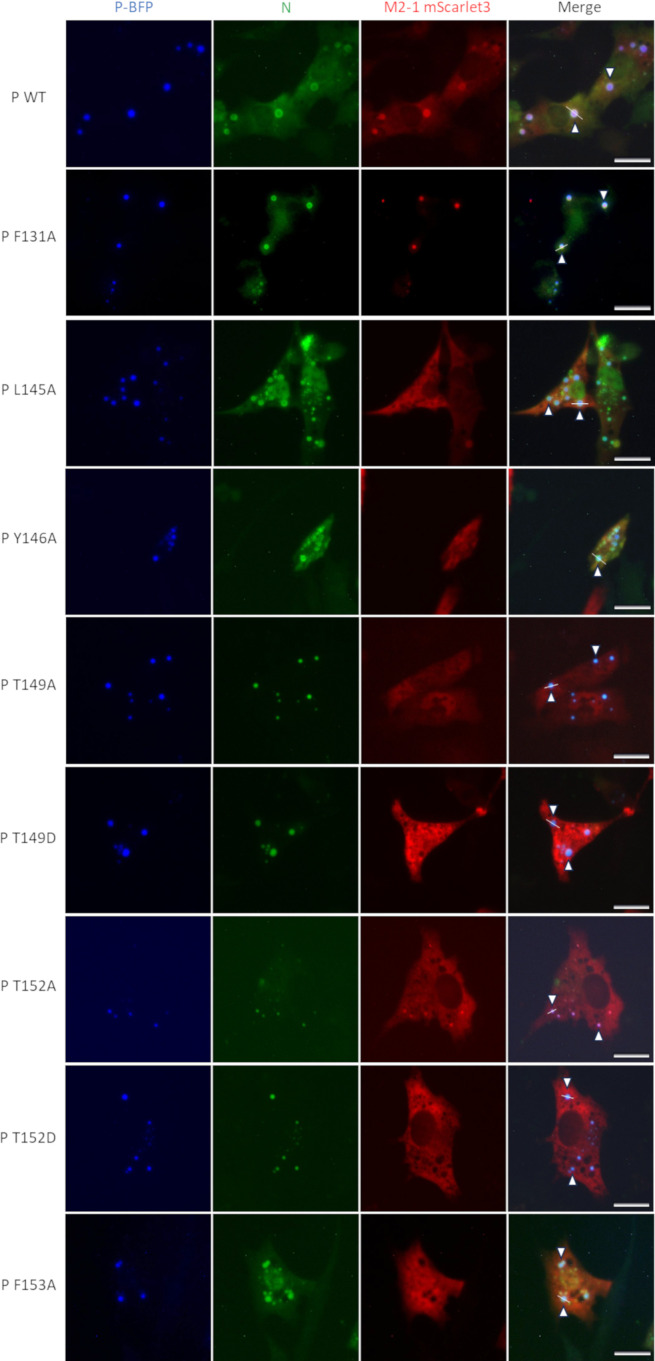
Effect of point mutations in the M2-1 binding site of P on protein localization in living cells. BSRT7 cells were transfected with plasmids encoding the N, P-BFP (WT and variants), and M2-1-Scarlet3 proteins as indicated. Twenty-four hours post-transfection, cells were fixed and labeled with a rabbit polyclonal anti-N antibody and detected with a secondary goat anti-rabbit antibody. The colocalization of proteins in IBs was analyzed using fluorescence microscopy. Quantification of red, green, and blue fluorescence (see Fig. S6) was performed on white bars placed on different IBs. Scale bars, 20 µm.

We then used a complementary technique to validate the functionality of the PP1 and M2-1 putative binding sites on SOV P. An eGFP-PP1 expression vector (31) was used for this purpose. Because we had no antibody against M2-1, a M2-1-Scarlet3 fusion synthetic construct was cloned into a pCite vector and co-transfected with N and P expression vectors in BSRT7 cells. As shown in Fig. 7, eGFP-PP1 was concentrated in IB-like structures when co-expressed with N and P-BFP, but not when a F131A variant of the P protein was used. M2-1-Scarlet3 was also found to be associated with IBs when co-expressed with N and P-BFP (WT and F131A variant) (Fig. 8; Fig. S5). However, for the P variants L145A, Y146A, T149A, T149D, and F153A, M2-1 was excluded from IBs and scattered in the cytosol (Fig. 8; Fig. S6). For threonine 152, which can be potentially phosphorylated, comparison between the phosphomimetic variants T152A and T152D showed that M2-1 was excluded from the IBs for T152D substitution, suggesting that phosphorylation of this residue could modulate its interaction with P. These results strongly suggest that PP1 and M2-1 are both recruited to SOV viral factories via P and that the residue F131 of P plays a critical role in the P-PP1 interaction.

**Fig 9:**
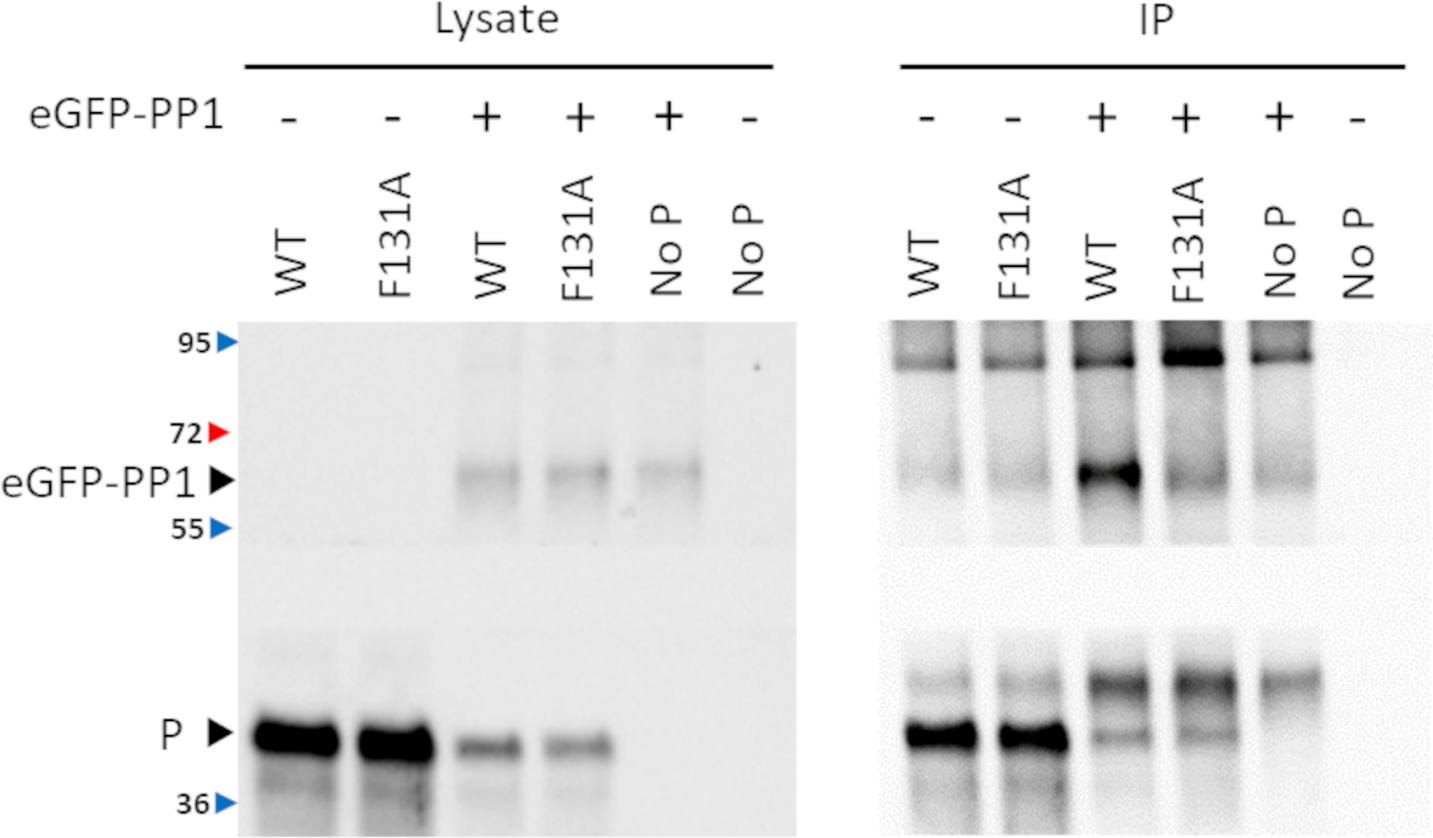
Effect of point mutation in the PP1 binding site of P on protein interactions in cells. HEK 293T cells were transfected with plasmids encoding Flag-P (WT and variant F131A) and eGFP-PP1, and immunoprecipitation was performed 24 h post-transfection using an anti-P antibody followed by Western blotting. Proteins were detected using anti-P and anti-GFP (eGFP-PP1) antibodies.

To confirm that the cellular phosphatase PP1 interacts with P, we performed immunoprecipitation after co-transfecting BSRT7 cells with plasmids encoding eGFP-PP1 and SOV P (WT and variant F131A) and by precipitating with an anti-Flag monoclonal antibody. The precipitated complexes were analyzed by Western blot using anti-P polyclonal serum and anti-GFP antibody. The presence of PP1 was revealed in the precipitated products using WT P (Fig. 9). Even though the expression level of P protein was lower for P + eGFP-PP1 co-transfection (compare lanes 3 and 4 to the first two lanes), with the P F131A variant, the PP1 signal was much weaker (Fig. 9), emphasizing the role of this residue in this interaction.

## DISCUSSION

The swine orthopneumovirus SOV was recently discovered and detected in pig farms in the USA (2), France (4), Germany (6), Spain (5), Korea (3), and Sweden (7). However, since this virus has not been isolated, it has not been possible to perform experimental infections, and it is still unknown whether this virus is pathogenic by itself. SOV could be intrinsically pathogenic or opportunistic, with potentially additional negative effects on pigs’ health in the case of co-infection with other porcine viruses, such as influenza, corona-, arteri-, respiro-, or circoviruses, as well as bacteria. To clarify this point, it is necessary to obtain infectious particles. Using the two Korean “complete” sequences KSOV-2201 and KSOV-2202 as well as other sequences of SOV-57, two PVM strains and one CPV strain, we established a functional minigenome system. By comparing the sequences of the different viruses and strains, we established consensus and functional sequences for SOV minigenome and polymerase complex that differed from the deposited KSOV-2201 sequence. Therefore, we conclude that the published KSOV-2201 was not optimum and contained errors, particularly in the Leader region. During the preparation of this manuscipt, a new study was published showing the presence of SOV in Sweden (7). Two complete sequences of SOV were published. Alignment of the 5′ end of the genomic cDNA sequences of these viruses with other sequences confirmed that the Leader2 18 first nucleotides we used for the minigenome system were identical to these two sequences, including a T at the 5′ end (Fig. S4). Notably, the presence of an A at the 3′ end of the genome (corresponding to the T at the 5′ end of the genomic cDNA) is unique to SOV and CPV and is not found in PVM or other pneumoviruses that generally terminate with a U (or A at the 5′ end of the genomic cDNA sequence). The next step will consist of developing a complete reverse genetics system using the same strategy, allowing the rescue of infectious particles and the performance of experimental infection of piglets. If this virus is pathogenic, whether alone or in co-infection, vaccine approaches could be developed rapidly, thanks to HRSV experience and based on artificially stabilized prefusion F proteins.

Similar to PVM, SOV has been classified in the *Pneumoviridae* family and the *Orthopneumovirus* genus, at least in part, because of the presence of NS genes at the 3′ end of the genome, which are absent from the members of the *Metapneumovirus* genus. However, previous phylogenetic analysis and sequence comparisons between the members of the *Pneumoviridae* family have shown that SOV, PVM, and CPV constitute a separate genogroup, sharing 90%–98% identity at the nucleotide sequence level (2, 3, 7). If we compare the sequences at the protein level, the homologies between SOV and PVM are even stronger, with, for example, 98% identity for the N protein compared to 59% between SOV and HRSV. In this study, we show that the RdRp complex of PVM, but not of HRSV, can rescue the SOV minigenome, reinforcing the idea that SOV, PVM, and CPV constitute a genus distinct from *Orthopneumovirus*, and obviously from *Metapneumovirus*. Thus, we suggest revisiting the classification of SOV and PVM in the *Pneumoviridae* family, which could be grouped in a new genus, such as *Parapneumovirus*, if validated by phylogenetics experts and the ICTV. SOV could also be changed to SPV for *Swine Pneumovirus* or *Swine Parapneumovirus.*

Using our functional minigenome system, we began to characterize the SOV RdRp polymerase complex. As discussed above, sequence comparison between SOV and other pneumoviruses showed that it is closely related to PVM and CPV but less so to HRSV and BRSV. Thus, despite this, our aim was to determine whether SOV RdRp has functional similarities with RSV. First, we investigated whether M2-1 is essential for viral transcription, as shown previously for HRSV and PVM. Using a bicistronic minigenome, we found that the transcription of both genes was abrogated in the absence of M2-1. These results were similar to those obtained with the PVM. Although the molecular mechanisms of PVM M2-1 on transcription have not been studied in detail, extensive studies have been performed with HRSV and, to a lesser extent, with HMPV, for which the role of M2-1 in transcription is unclear. For HRSV, it has been determined that M2-1 is not involved in replication, transcription initiation and termination, polyadenylation, or RNA capping (33). It is not required for the transcription of short mRNAs, but it is needed for the transcription of mRNAs longer than 500 nt. Therefore, its function was, thus, considered a transcription elongation factor (14, 15). It was further shown that HRSV M2-1 binds to RNAs in a competitive manner with the P protein (13, 32), co-localizes with HRSV mRNAs in inclusion body subcompartments called IBAGs (20), and has a higher affinity for A- or AU-rich sequences, such as antisense gene end signals located at the end of viral mRNAs (8, 34). HRSV M2-1 also associates with AU-rich cellular mRNAs in infected cells (16, 33). In our minigenome construct, the Gaussia gene is located at the 3′ end of the construct under the control of the leader region and the first gene start signal. This gene is ~600 nt in length and thus could require the help of M2-1 for proper elongation, as shown for HRSV. Further investigations are necessary to clarify the role of SOV in transcription, its RNA affinities, and other similarities or differences with HRSV.

Sequence comparison between SOV and HRSV P proteins revealed the presence of similar sequences in regions previously identified as cellular PP1 and viral M2-1 protein-binding motifs. This was confirmed by site-directed mutagenesis and *in cellula* assays, as minigenome activity was strongly reduced by some critical substitutions at both sites. Using cell transfections and immunofluorescence, we observed that the loss of minigenome activity was associated with the loss of PP1 and M2-1 localization in pseudo-IBs. In particular, T149D and T152D phosphomimetic substitutions on P had a drastic effect on M2-1 localization in IBs and minigenome activity, indicating that a cyclic phosphorylation/dephosphorylation of these residues can play a role in the regulation of the formation of a P-M2-1 complex and SOV transcription, as it was described for HRSV (31). In conclusion, despite the marked sequence divergence between SOV and RSV, these viruses show some similarities at the functional level.

## MATERIALS AND METHODS

### Design of a minigenome system for SOV

We used a synthetic sequence corresponding to the Korean strain KSOV-2201 (GenBank accession number: OR701947.1). Synthetic sequences coding for N, P, M2-1, and L genes (Gene Universal, Newark, USA) were cloned into pCite vectors (13, 35, 36) under the control of a T7 promoter. A minigenome was designed based on the KSOV-2201 sequence. It contains the Leader and Trailer sequences, and two ORF coding for Gaussia and Firefly luciferases, respectively, under the control of gene start (GS) and gene end (GE) sequences of SOV, which are identical to the GS and GE sequences of PVM (see Fig. 1; Fig. S1). The T7 promoter is located upstream of the trailer sequence such that a negative sense minigenome RNA is transcribed with three supplementary G residues at the 5′ end. The hepatitis delta virus (HDV) ribozyme sequence is fused to the last nucleotide of the leader sequence to achieve correct cleavage at the 3′ end. The different minigenomes containing either the original KSOV-2201 (pMiniG0) or modified leader sequences (pMiniG1 and pMiniG2) were synthesized by GeneCust (Boynes, France) and cloned into a pUC57 plasmid.

### Minigenome assays

BSRT7/5 cells constitutively expressing the phage T7 RNA polymerase were grown in Dulbecco’s modified Eagle’s medium (Lonza) supplemented with 10% fetal calf serum (FCS), 2 mM glutamine, and 1% penicillin-streptomycin. BSRT7/5 cells at 90% confluence in 48-well dishes were transfected with a plasmid mixture containing 125 ng of pMiniG, 125 ng of pN, 125 ng of pP, 125 ng of pM2-1, 62.5 ng of pL, and 37.5 ng of pCMV-b-Gal (Clontech), to normalize transfection efficiencies. Cells were transfected using the Lipofectamine 2000 reagent (Invitrogen, Cergy-Pontoise, France) in Opti-MEM medium (Gibco), as described by the manufacturer. The cells were harvested at 24 h post-transfection, and the Firefly and Gaussia luciferase activities were determined for each cell lysate using the Luciferase Assay (Promega) and Renilla Luciferase Assay System (Promega), respectively, quantified with an Infinite 200 Pro microplate reader (Tecan, Männedorf, Switzerland) and normalized based on beta-galactosidase expression and on the value obtained for transfected and untreated cells. The transfections were performed in triplicate, and each independent transfection experiment was performed at least three times. Statistical analyses were performed with Prism 8.0.1 (GraphPad Software, Inc.) and were done by unpaired *t*-test with Welch’s correction. A *P* < 0.05 was considered to be statistically significant.

Point mutations were introduced into the P sequence by site-directed mutagenesis using the Q5 site-directed mutagenesis kit (New England Biolabs). The HRSV Minigenome system has been described previously (8). Plasmids encoding for the PVM RdRp were described previously (10).

### Generation of N and P rabbit antisera

The recombinant SOV N protein was produced in *E. coli* and purified as previously described (4). Polyclonal antiserum was prepared by immunizing a rabbit three times at 2–4 weeks intervals using purified N proteins (100 mg) for each immunization. All immunizations were administered subcutaneously and emulsified with Gerbu P adjuvant (Gerbu Biotechnik, Germany) at a 1:1 (vol/vol) ratio with the solubilized antigen. The rabbit was bled 14 days after the third immunization. A codon-optimized SOV P sequence adapted for *E. coli* expression was synthesized by GenScript and cloned into the pGEX-4T-3 vector. The protein was purified as described previously (4) and the rabbit immunization protocol complied with EU legislation (authorization APAFIS #47410-2024013017438011 v4 accorded by the French Ministry of Higher Education and Research).

### Insertion of fluorescent tags in P and M2-1 proteins

To find a place in the P protein where the insertion of a fluorescent tag should not or poorly affect its functions, we used sequence analysis, structure prediction, and sequence alignments between SOV strains, PVM, and CPV (see Fig. S2 and S3). The SOV P sequence (GenBank accession number WPS68790.1) was submitted to hydrophobic cluster analysis using the DRAWHCA program (https://mobyle.rpbs.univ-paris-diderot.fr/cgi-bin/portal.py#forms::HCA), the Predictor Of Naturally Disordered Regions (PONDR) server ( https://www.pondr.com/) using VLXT method, and AlphaFold3 (https://alphafoldserver.com/welcome).

The region 61-RVAANLTNPSVPPSTPPSIPP-81 appeared as a highly disordered and less conserved region, making it a good candidate for tag insertion (Fig. S3). An AgeI restriction site was inserted between residues 75-76 of P using the Q5 Site-Directed Mutagenesis Kit (NEB) and the following primers: P-Age1-forward: GGTGCTGGGTGGCACAGAGGG, P-Age1-reverse: GGTCCTCCATCCATCCCTCCC. Then, PBFP was then amplified from the pTagBFP-Tubulin vector (Evrogen) using the following primers: BFP-Age1-forward: GCCACCCAGCACCGGATGAGCGAGCTGATTAAGGAGAAC; BFP-Age1-reverse: TGGATGGAGGACCGGTCGAGATCTGAGTCCGGAATTAA and inserted at AgeI site by using In-Fusion HD Cloning Kit (Clontech).

Based on what was done with HRSV (31), the plasmid pM2-1-mScarlet3 was obtained by subcloning the sequence encoding mScarlet3 (37) in frame at the 3′ end of the M2-1 gene with the following primers: Scarlet3-XhoI-forward: GAGAACAGAACTCGAGATGGATAGCACCGAGGCAGTGATC; Scarlet3-XhoI-reverse:AAGCTAGAGGCTCGATTAGGAGCCACCGGAGCCGCCGG.The amplicon containing the mScarlet3 sequence was inserted at the XhoI site using the In-Fusion HD Cloning Kit.

### Fluorescence microscopy

BSRT7/5 cells grown on coverslips were transfected with pN, pM2-1-mScarlet3, and pP-BFP (WT or variants) using Lipofectamine2000 (Invitrogen). At 24 h post-transfection, samples were fixed in 4% paraformaldehyde (PFA) for 30 min and permeabilized in PBS containing 0.1% Triton X-100 and 3% BSA. The coverslips were incubated for 1 h at room temperature with a rabbit anti-N antibody for N detection, washed, and then incubated for an additional hour with Alexa Fluor 488 goat anti-rabbit IgG (Invitrogen). Coverslips were mounted using ProLong Gold Antifade reagent (Life Technologies). Cells were observed using a Nikon TE200 inverted microscope equipped with a Photometrics CoolSNAP ES2 camera. Images were processed using MetaVue software (Molecular Devices). RGB profiles were done using ImageJ software.

### Immunoblotting

Cells were lysed for 30 min at 4°C in lysis buffer (20 mMTris [pH 7.4], 150 mMNaCl, 0.1% Triton X-100) supplemented with a complete protease inhibitor cocktail (Roche). Cell lysates were centrifuged for 10 min at 10,000 × *g*, and the supernatants were recovered, mixed with Laemmli buffer, and boiled. Proteins were resolved by SDS-PAGE and transferred onto nitrocellulose membranes. The membranes were incubated in blocking solution (1× PBS, 0.05% Tween 20 supplemented with 5% milk) for 1 h. Blots were incubated with primary antibodies in blocking solution: rabbit anti-P antiserum, mouse monoclonal anti-α-tubulin antibody (Sigma), and rabbit anti-GFP antibody (Invitrogen). The membranes were rinsed with PBS containing 0.05% Tween 20 and incubated for 1 h with the appropriate HRP-conjugated secondary antibodies diluted in blocking solution. The membranes were rinsed, and immunodetection was performed by using an enhanced chemiluminescence (ECL) substrate (BioRad, France).

### Coimmunoprecipitation assays

HEK 293T cells (ATCC CRL-3216) were co-transfected with pCDNA3-P (WT and variant, codon-optimized P for mammalian expression by GeneCust, Boynes, France) and peGFP-PP1. After 24 h, the transfected cells were lysed for 30 min at 4°C in ice-cold lysis buffer (20 mM Tris HCl [pH 7.4], 150 mM NaCl, 0.1% Triton X100 and 15% glycerol) with a complete protease inhibitor cocktail (Roche). Cell lysates were centrifuged at 4°C for 10 min at 10,000 *g* and incubated overnight at 4°C with a mouse anti-Flag monoclonal antibody (Sigma-Aldrich cl. M5) coupled to Protein G beads (Invitrogen). The beads were then washed three times with lysis buffer and one time with PBS, proteins were boiled in Laemmli buffer for 5 min and samples were subjected to SDS-PAGE and immunoblotting as described above.

## Data Availability

All data generated and analyzed during this study are described in the article. The intermediate and supporting data are available from the corresponding author upon reasonable request.

## SUPPLEMENTAL MATERIAL

Supplemental figures jvi.00363-26-s0001.pdf Fig. S1 to S6.

ASM does not own the copyrights to Supplemental Material that may be linked to, or accessed through, an article. The authors have granted ASM a non-exclusive, world-wide license to publish the Supplemental Material files. Please contact the corresponding author directly for reuse.
