# Supplementary material for: Functional minigenome system reveals polymerase features of swine orthopneumovirus
Source: J Virol. 2026 May 19;100(6):e00363-26. doi: 10.1128/jvi.00363-26 (PMC13288936; doi:10.1128/jvi.00363-26)
Supplement: Supplemental figures — Fig. S1 to S6. [file jvi.00363-26-s0001.pdf]

A

5’
caaaaaacccctcaagacccgtttagaggccccaaggggttatgctaggaggaggagAGATCTctcccttagccatccgagtggacgacgtcctccttcg
gatgcccaggtcggaccgcgaggaggtggagatgccatgccgacccacccgaaaaaaaacataacacaactatcaccgaaaaaagataggacaagtagga
attgctagtcctaaagaccaggatcATGGGAGTCAAAGTTCTGTTTGCCCTGATCTGCATCGCTGTGGCCGAGGCCAAGCCCACCGAGAACAACGAAGAC
TTCAACATCGTGGCCGTGGCCAGCAACTTCGCGACCACGGATCTCGATGCTGACCGCGGGAAGTTGCCCGGCAAGAAGCTGCCGCTGGAGGTGCTCAAAG
AGATGGAAGCCAATGCCCGGAAAGCTGGCTGCACCAGGGGCTGTCTGATCTGCCTGTCCCACATCAAGTGCACGCCCAAGATGAAGAAGTTCATCCCAGG
ACGCTGCCACACCTACGAAGGCGACAAAGAGTCCGCACAGGGCGGCATAGGCGAGGCGATCGTCGACATTCCTGAGATTCCTGGGTTCAAGGACTTGGAG
CCGATGGAGCAGTTCATCGCACAGGTCGATCTGTGTGTGGACTGCACAACTGGCTGCCTCAAAGGGCTTGCCAACGTGCAGTGTTCTGACCTGCTCAAGA
AGTGGCTGCCGCAACGCTGTGCGACCTTTGCCAGCAAGATCCAGGGCCAGGTGGACAAGATCAAGGGGGCCGGTGGTGACTAAtagttatagaaaattat
taggataaataATGGAAGACGCCAAAAACATAAAGAAAGGCCCGGCGCCATTCTATCCTCTAGAGGATGGAACCGCTGGAGAGCAACTGCATAAGGCTAT
GAAGAGATACGCCCTGGTTCCTGGAACAATTGCTTTTACAGATGCACATATCGAGGTGAACATCACGTACGCGGAATACTTCGAAATGTCCGTTCGGTTG
GCAGAAGCTATGAAACGATATGGGCTGAATACAAATCACAGAATCGTCGTATGCAGTGAAAACTCTCTTCAATTCTTTATGCCGGTGTTGGGCGCGTTAT
TTATCGGAGTTGCAGTTGCGCCCGCGAACGACATTTATAATGAACGTGAATTGCTCAACAGTATGAACATTTCGCAGCCTACCGTAGTGTTTGTTTCCAA
AAAGGGGTTGCAAAAAATTTTGAACGTGCAAAAAAAATTACCAATAATCCAGAAAATTATTATCATGGATTCTAAAACGGATTACCAGGGATTTCAGTCG
ATGTACACGTTCGTCACATCTCATCTACCTCCCGGTTTTAATGAATACGATTTTGTACCAGAGTCCTTTGATCGTGACAAAACAATTGCACTGATAATGA
ATTCCTCTGGATCTACTGGGTTACCTAAGGGTGTGGCCCTTCCGCATAGAACTGCCTGCGTCAGATTCTCGCATGCCAGAGATCCTATTTTTGGCAATCA
AATCATTCCGGATACTGCGATTTTAAGTGTTGTTCCATTCCATCACGGTTTTGGAATGTTTACTACACTCGGATATTTGATATGTGGATTTCGAGTCGTC
TTAATGTATAGATTTGAAGAAGAGCTGTTTTTACGATCCCTTCAGGATTACAAAATTCAAAGTGCGTTGCTAGTACCAACCCTATTTTCATTCTTCGCCA
AAAGCACTCTGATTGACAAATACGATTTATCTAATTTACACGAAATTGCTTCTGGGGGCGCACCTCTTTCGAAAGAAGTCGGGGAAGCGGTTGCAAAACG
CTTCCATCTTCCAGGGATACGACAAGGATATGGGCTCACTGAGACTACATCAGCTATTCTGATTACACCCGAGGGGGATGATAAACCGGGCGCGGTCGGT
AAAGTTGTTCCATTTTTTGAAGCGAAGGTTGTGGATCTGGATACCGGGAAAACGCTGGGCGTTAATCAGAGAGGCGAATTATGTGTCAGAGGACCTATGA
TTATGTCCGGTTATGTAAACAATCCGGAAGCGACCAACGCCTTGATTGACAAGGATGGATGGCTACATTCTGGAGACATAGCTTACTGGGACGAAGACGA
ACACTTCTTCATAGTTGACCGCTTGAAGTCTTTAATTAAATACAAAGGATATCAGGTGGCCCCCGCTGAATTGGAATCGATATTGTTACAACACCCCAAC
ATCTTCGACGCGGGCGTGGCAGGTCTTCCCGACGATGACGCCGGTGAACTTCCCGCCGCCGTTGTTGTTTTGGAGCACGGAAAGACGATGACGGAAAAAG
AGATCGTGGATTACGTCGCCAGTCAAGTAACAACCGCGAAAAAGTTGCGCGGAGGAGTTGTGTTTGTGGACGAAGTACCGAAAGGTCTTACCGGAAAACT
CGACGCAAGAAAAATCAGAGAGATCCTCATAAAGGCCAAGAAGGGCGGAAAGTCCAAATTGTAActtgatgagttaacactaacatgtcttctttataac
ttttcttgtttaaccactcttagcttagacatgtgagatgatcttaaacctagctgtacctcttctatttggaactgattgtagctttagtagctctgtc
cttcttgatttctgatcagcagcaagcgtattgttattttaatttgtatatagttaacaaaaaaccgatatcttaagggttgtgatcatagctctttcat
Aattattgttagttaaactattgtgtttgacttcttttgggtattttttcccgtcctatagtgagtcgtatta – 3’

B

5’
caaaaaacccctcaagacccgtttagaggccccaaggggttatgctaggaggaggagAGATCTctcccttagccatccgagtggacgacgtcctccttcg
gatgcccaggtcggaccgcgaggaggtggagatgccatgccgaccctacgcgaaaaaatgcataacaaaactatcaacctgaaaaagttaggacaagtag
gaattgctagtcctaaagaccaggatcATGGGAGTCAAAGTTCTGTTTGCCCTGATCTGCATCGCTGTGGCCGAGGCCAAGCCCACCGAGAACAACGAAG
ACTTCAACATCGTGGCCGTGGCCAGCAACTTCGCGACCACGGATCTCGATGCTGACCGCGGGAAGTTGCCCGGCAAGAAGCTGCCGCTGGAGGTGCTCAA
AGAGATGGAAGCCAATGCCCGGAAAGCTGGCTGCACCAGGGGCTGTCTGATCTGCCTGTCCCACATCAAGTGCACGCCCAAGATGAAGAAGTTCATCCCA
GGACGCTGCCACACCTACGAAGGCGACAAAGAGTCCGCACAGGGCGGCATAGGCGAGGCGATCGTCGACATTCCTGAGATTCCTGGGTTCAAGGACTTGG
AGCCGATGGAGCAGTTCATCGCACAGGTCGATCTGTGTGTGGACTGCACAACTGGCTGCCTCAAAGGGCTTGCCAACGTGCAGTGTTCTGACCTGCTCAA
GAAGTGGCTGCCGCAACGCTGTGCGACCTTTGCCAGCAAGATCCAGGGCCAGGTGGACAAGATCAAGGGGGCCGGTGGTGACTAAtagttatagaaaatt
attaggataaataATGGAAGACGCCAAAAACATAAAGAAAGGCCCGGCGCCATTCTATCCTCTAGAGGATGGAACCGCTGGAGAGCAACTGCATAAGGCT
ATGAAGAGATACGCCCTGGTTCCTGGAACAATTGCTTTTACAGATGCACATATCGAGGTGAACATCACGTACGCGGAATACTTCGAAATGTCCGTTCGGT
TGGCAGAAGCTATGAAACGATATGGGCTGAATACAAATCACAGAATCGTCGTATGCAGTGAAAACTCTCTTCAATTCTTTATGCCGGTGTTGGGCGCGTT
ATTTATCGGAGTTGCAGTTGCGCCCGCGAACGACATTTATAATGAACGTGAATTGCTCAACAGTATGAACATTTCGCAGCCTACCGTAGTGTTTGTTTCC
AAAAAGGGGTTGCAAAAAATTTTGAACGTGCAAAAAAAATTACCAATAATCCAGAAAATTATTATCATGGATTCTAAAACGGATTACCAGGGATTTCAGT
CGATGTACACGTTCGTCACATCTCATCTACCTCCCGGTTTTAATGAATACGATTTTGTACCAGAGTCCTTTGATCGTGACAAAACAATTGCACTGATAAT
GAATTCCTCTGGATCTACTGGGTTACCTAAGGGTGTGGCCCTTCCGCATAGAACTGCCTGCGTCAGATTCTCGCATGCCAGAGATCCTATTTTTGGCAAT
CAAATCATTCCGGATACTGCGATTTTAAGTGTTGTTCCATTCCATCACGGTTTTGGAATGTTTACTACACTCGGATATTTGATATGTGGATTTCGAGTCG
TCTTAATGTATAGATTTGAAGAAGAGCTGTTTTTACGATCCCTTCAGGATTACAAAATTCAAAGTGCGTTGCTAGTACCAACCCTATTTTCATTCTTCGC
CAAAAGCACTCTGATTGACAAATACGATTTATCTAATTTACACGAAATTGCTTCTGGGGGCGCACCTCTTTCGAAAGAAGTCGGGGAAGCGGTTGCAAAA
CGCTTCCATCTTCCAGGGATACGACAAGGATATGGGCTCACTGAGACTACATCAGCTATTCTGATTACACCCGAGGGGGATGATAAACCGGGCGCGGTCG
GTAAAGTTGTTCCATTTTTTGAAGCGAAGGTTGTGGATCTGGATACCGGGAAAACGCTGGGCGTTAATCAGAGAGGCGAATTATGTGTCAGAGGACCTAT
GATTATGTCCGGTTATGTAAACAATCCGGAAGCGACCAACGCCTTGATTGACAAGGATGGATGGCTACATTCTGGAGACATAGCTTACTGGGACGAAGAC
GAACACTTCTTCATAGTTGACCGCTTGAAGTCTTTAATTAAATACAAAGGATATCAGGTGGCCCCCGCTGAATTGGAATCGATATTGTTACAACACCCCA
ACATCTTCGACGCGGGCGTGGCAGGTCTTCCCGACGATGACGCCGGTGAACTTCCCGCCGCCGTTGTTGTTTTGGAGCACGGAAAGACGATGACGGAAAA
AGAGATCGTGGATTACGTCGCCAGTCAAGTAACAACCGCGAAAAAGTTGCGCGGAGGAGTTGTGTTTGTGGACGAAGTACCGAAAGGTCTTACCGGAAAA
CTCGACGCAAGAAAAATCAGAGAGATCCTCATAAAGGCCAAGAAGGGCGGAAAGTCCAAATTGTAActtgatgagttaacactaacatgtcttctttata
acttttcttgtttaaccactcttagcttagacatgtgagatgatcttaaacctagctgtacctcttctatttggaactgattgtagctttagtagctctg
tccttcttgatttctgatcagcagcaagcgtattgttattttaatttgtatatagttaacaaaaaaccgatatcttaagggttgtgatcatagctctttc
Ataattattgttagttaaactattgtgtttgacttcttttgggtattttttcccgtcctatagtgagtcgtatta – 3’

SUPPLEMENTARY FIG 1 Nucleotide sequence of the SOV Minigenome constructs. (A) Minigenome with the Leader sequence of KSOV-2201 based on the published sequence. **(B)** Minigenome with the Leader2 sequence. The T7 promoter at the 3’ end is in blue letters. The Leader and Trailer sequences are highlighted in yellow and blue, respectively. The gene start (green letters) and gene end (red letters) signals are derived from KSOV-2201. The Gaussia and Firefly luciferases are represented by orange and magenta letters, respectively. ORFs are indicated by capital letters. The ribozyme and the T7 terminator a the 5’ end are in blue and red letters, respectively.

L

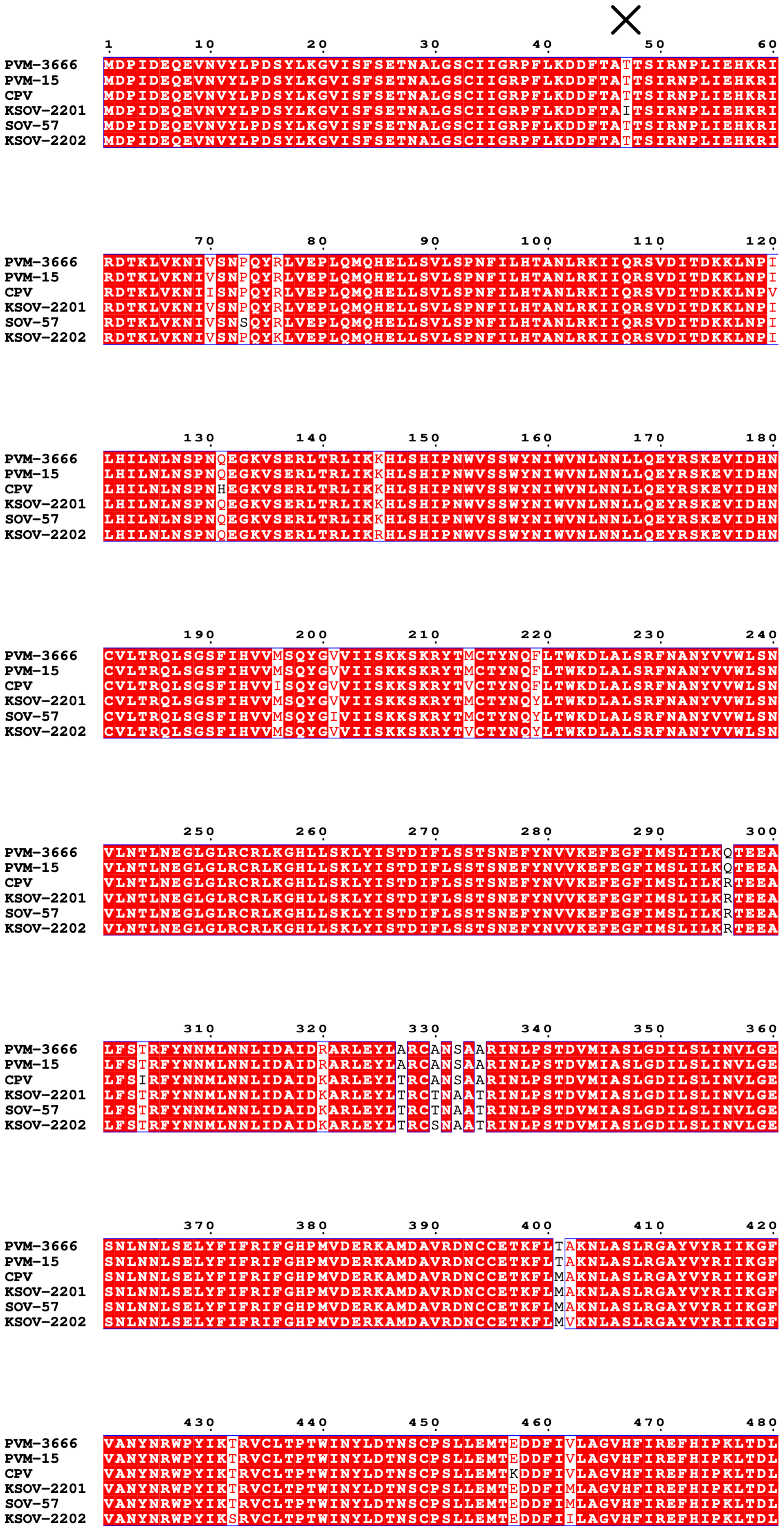
1 10 20 30 40 50 60
PVM-3666 MDPIDEQEVNVYLPDSYLKGVISFSETNALGSCIIGRPFLKDDFTATTSIRNPLIEHKRI
PVM-15 MDPIDEQEVNVYLPDSYLKGVISFSETNALGSCIIGRPFLKDDFTATTSIRNPLIEHKRI
CPV MDPIDEQEVNVYLPDSYLKGVISFSETNALGSCIIGRPFLKDDFTATTSIRNPLIEHKRI
KSOV-2201 MDPIDEQEVNVYLPDSYLKGVISFSETNALGSCIIGRPFLKDDFTAITSIRNPLIEHKRI
SOV-57 MDPIDEQEVNVYLPDSYLKGVISFSETNALGSCIIGRPFLKDDFTATTSIRNPLIEHKRI
KSOV-2202 MDPIDEQEVNVYLPDSYLKGVISFSETNALGSCIIGRPFLKDDFTATTSIRNPLIEHKRI
70 80 90 100 110 120
PVM-3666 RDTKLVKNIVSNPQYRLVEPLQMQHELLSVLSPNFILHTANLRKIIQRSVDITDKKLNPI
PVM-15 RDTKLVKNIVSNPQYRLVEPLQMQHELLSVLSPNFILHTANLRKIIQRSVDITDKKLNPI
CPV RDTKLVKNIISNPQYRLVEPLQMQHELLSVLSPNFILHTANLRKIIQRSVDITDKKLNPV
KSOV-2201 RDTKLVKNIVSNPQYRLVEPLQMQHELLSVLSPNFILHTANLRKIIQRSVDITDKKLNPI
SOV-57 RDTKLVKNIVSNSQYRLVEPLQMQHELLSVLSPNFILHTANLRKIIQRSVDITDKKLNPI
KSOV-2202 RDTKLVKNIVSNPQYKLVEPLQMQHELLSVLSPNFILHTANLRKIIQRSVDITDKKLNPI
130 140 150 160 170 180
PVM-3666 LHILNLNSPNQEGKVSERLTRLIKKHLSHIPNWVSSWYNIWVNLNNLLQEYRSKEVIDHN
PVM-15 LHILNLNSPNQEGKVSERLTRLIKKHLSHIPNWVSSWYNIWVNLNNLLQEYRSKEVIDHN
CPV LHILNLNSPNHEGKVSERLTRLIKKHLSHIPNWVSSWYNIWVNLNNLLQEYRSKEVIDHN
KSOV-2201 LHILNLNSPNQEGKVSERLTRLIKKHLSHIPNWVSSWYNIWVNLNNLLQEYRSKEVIDHN
SOV-57 LHILNLNSPNQEGKVSERLTRLIKKHLSHIPNWVSSWYNIWVNLNNLLQEYRSKEVIDHN
KSOV-2202 LHILNLNSPNQEGKVSERLTRLIKRHLSHIPNWVSSWYNIWVNLNNLLQEYRSKEVIDHN
190 200 210 220 230 240
PVM-3666 CVLTRQLSGSFIHVVMSQYGVVIISKKSKRYTMCTYNQFLTWKDLALSRFNANYVVWLSN
PVM-15 CVLTRQLSGSFIHVVMSQYGVVIISKKSKRYTMCTYNQFLTWKDLALSRFNANYVVWLSN
CPV CVLTRQLSGSFIHVVISQYGVVIISKKSKRYTVCTYNQFLTWKDLALSRFNANYVVWLSN
KSOV-2201 CVLTRQLSGSFIHVVMSQYGVVIISKKSKRYTMCTYNQYLTWKDLALSRFNANYVVWLSN
SOV-57 CVLTRQLSGSFIHVVMSQYGIVIISKKSKRYTMCTYNQYLTWKDLALSRFNANYVVWLSN
KSOV-2202 CVLTRQLSGSFIHVVMSQYGVVIISKKSKRYTVCTYNQYLTWKDLALSRFNANYVVWLSN
250 260 270 280 290 300
PVM-3666 VLNTLNEGLGLRCRLKGHLLSKLYISTDIFLSSTSNEFYNVVKEFEGFIMSLILKQTEEA
PVM-15 VLNTLNEGLGLRCRLKGHLLSKLYISTDIFLSSTSNEFYNVVKEFEGFIMSLILKQTEEA
CPV VLNTLNEGLGLRCRLKGHLLSKLYISTDIFLSSTSNEFYNVVKEFEGFIMSLILKRTEEA
KSOV-2201 VLNTLNEGLGLRCRLKGHLLSKLYISTDIFLSSTSNEFYNVVKEFEGFIMSLILKRTEEA
SOV-57 VLNTLNEGLGLRCRLKGHLLSKLYISTDIFLSSTSNEFYNVVKEFEGFIMSLILKRTEEA
KSOV-2202 VLNTLNEGLGLRCRLKGHLLSKLYISTDIFLSSTSNEFYNVVKEFEGFIMSLILKRTEEA
310 320 330 340 350 360
PVM-3666 LFSTRFYNNMLNNLIDAIDRARLEYLARCANSAARINLPSTDVMIASLGDILSLINVLGE
PVM-15 LFSTRFYNNMLNNLIDAIDRARLEYLARCANSAARINLPSTDVMIASLGDILSLINVLGE
CPV LFSIRFYNNMLNNLIDAIDKARLEYLTRCANSAARINLPSTDVMIASLGDILSLINVLGE
KSOV-2201 LFSTRFYNNMLNNLIDAIDKARLEYLTRCTNAATRINLPSTDVMIASLGDILSLINVLGE
SOV-57 LFSTRFYNNMLNNLIDAIDKARLEYLTRCTNAATRINLPSTDVMIASLGDILSLINVLGE
KSOV-2202 LFSTRFYNNMLNNLIDAIDKARLEYLTRCSNAATRINLPSTDVMIASLGDILSLINVLGE
370 380 390 400 410 420
PVM-3666 SNLNNLSELYFIFRIFGHPMVDERKAMDAVRDNCCETKFLTAKNLASLRGAYVYRIIKGF
PVM-15 SNLNNLSELYFIFRIFGHPMVDERKAMDAVRDNCCETKFLTAKNLASLRGAYVYRIIKGF
CPV SNLNNLSELYFIFRIFGHPMVDERKAMDAVRDNCCETKFLMAKNLASLRGAYVYRIIKGF
KSOV-2201 SNLNNLSELYFIFRIFGHPMVDERKAMDAVRDNCCETKFLMAKNLASLRGAYVYRIIKGF
SOV-57 SNLNNLSELYFIFRIFGHPMVDERKAMDAVRDNCCETKFLMAKNLASLRGAYVYRIIKGF
KSOV-2202 SNLNNLSELYFIFRIFGHPMVDERKAMDAVRDNCCETKFLMVKNLASLRGAYVYRIIKGF
430 440 450 460 470 480
PVM-3666 VANYNRWPYIKTRVCLTPTWINYLDTNSCPSLLEMTEDDFIVLAGVHFIREFHIPKLTDL
PVM-15 VANYNRWPYIKTRVCLTPTWINYLDTNSCPSLLEMTEDDFIVLAGVHFIREFHIPKLTDL
CPV VANYNRWPYIKTRVCLTPTWINYLDTNSCPSLLEMTKDDFIVLAGVHFIREFHIPKLTDL
KSOV-2201 VANYNRWPYIKTRVCLTPTWINYLDTNSCPSLLEMTEDDFIMLAGVHFIREFHIPKLTDL
SOV-57 VANYNRWPYIKTRVCLTPTWINYLDTNSCPSLLEMTEDDFIMLAGVHFIREFHIPKLTDL
KSOV-2202 VANYNRWPYIKSRVCLTPTWINYLDTNSCPSLLEMTEDDFIILAGVHFIREFHIPKLTDL

L

```
                   490       500       510       520       530       540
PVM-3666    EIILNDKAISPPKSLIWSCFPKNYIPQVIQDEYARRYCRAKAPLKTRRVLEFYLQDKDFK
PVM-15      EIILNDKAISPPKSLIWSCFPKNYIPQVIQDEYARRYCRAKAPLKTRRVLEFYLQDKDFK
CPV         EIILNDKAISPPKSLIWSCFPKNYIPQVIQDEYARRYCRAKAPLKTRRVLEFYLQDKDFK
KSOV-2201   EIILNDKAISPPKSLIWSCFPKNYIPQVIQDEYARRYYRAKAPLKTRRVLEFYLQDKDFK
SOV-57      EIILNDKAISPPKSLIWSCFPKNYIPQVIQDEYARRCYRAKAPLKTRRVLEFYLQDKDFK
KSOV-2202   EIILNDKAISPPKSLIWSCFPKNYIPQVIQDEYARRYYRAKAPLKTRRVLEFYLQDKDFK
```

```
                   550       560       570       580       590       600
PVM-3666    LDQLHRVVVNQDYLNDKEHIISLTGKERELGVGRMFAMQPGKQRQVQILAEKLLADNILQ
PVM-15      LDQLHRVVVNQDYLNDKEHIISLTGKERELGVGRMFAMQPGKQRQVQILAEKLLADNILQ
CPV         LDQLHRVVVNQDYLNDKEHIISLTGKERELSVGRMFAMQPGKQRQVQILAEKLLADNILQ
KSOV-2201   LDQLHRVVVSQDYLNDKEHIISLTGKERELSVGRMFAMQPGKQRQVQILAEKLLADNILQ
SOV-57      LDQLHRVVVSQDYLNDKEHIISLTGKERELSVGRMFAMQPGKQRQVQILAEKLLADNILQ
KSOV-2202   LDQLHRVVVSQDYLNDKEHIISLTGKERELSVGRMFAMQPGKQRQVQILAEKLLADNILQ
```

```
                   610       620       630       640       650       660
PVM-3666    FFPETLTRYGDLELQKILELKAGLSNKNDRSKDSYNNYISRCSLITDLSKFNQAFRYESS
PVM-15      FFPETLTRYGDLELQKILELKAGLSNKNDRSKDSYNNYISRCSLITDLSKFNQAFRYESS
CPV         FFPETLTRYGDLELQKILELKAGLSNKNDRSKDSYNNYISRCSLITDLSKFNQAFRYESS
KSOV-2201   FFPETLTRYGDLELQKILELKAGLSNKNGRSKDSYNNYISRCSLITDLSKFNQAFRYESS
SOV-57      FFPETLTRYGDLELQKILELKAGLSNKNGRSKDSYNNYISRCSLITDLSKFNQAFRYESS
KSOV-2202   FFPETLTRYGDLELQKILELKAGLSNKNGRSKDSYNNYISRCSLITDLSKFNQAFRYESS
```

```
                   670       680       690       700       710       720
PVM-3666    CVCSDLLDELHGTQSLFSWLHLTVPLTTIMCTYRHAPPDTGNNYNVDDIAEQSGLYRYHM
PVM-15      CVCSDLLDELHGTQSLFSWLHLTVPLTTIMCTYRHAPPDTGNNYNVDDIAEQSGLYRYHM
CPV         CVCSDLLDELHGTQSLFSWLHLTVPLTTIMCTYRHAPPDTGNNYNVDDIAEQSGLYRYHM
KSOV-2201   CVCSDLLDELHGTQSLFSWLHLTVPLTTIMCTYRHAPPDTGNNYDVDDIAEQSGLYRYHM
SOV-57      CVCSDLLDELHGTQSLFSWLHLTVPLTTIMCTYRHAPPDTGNNYDVDDIAEQSGLYRYHM
KSOV-2202   CVCSDLLDELHGTQSLFSWLHLTVPLTTIMCTYRHAPPDTGNNYDVDDIAEQSGLYRYHM
```

```
                   730       740       750       760       770       780
PVM-3666    GGIEGWCQKLWTTEAIALLDTVAVKGRFQLTSLINGDNQSIDISKPTRLGTRTQSEADYD
PVM-15      GGIEGWCQKLWTTEAIALLDTVAVKGRFQLTSLINGDNQSIDISKPTRLGTRTQSEADYD
CPV         GGIEGWCQKLWTTEAIALLDTVAVKGRFQLTSLINGDNQSIDISKPTRLGTRTQSEADYD
KSOV-2201   GGIEGWCQKLWTTEAIALLDTVAVKGRFQLTSLINGDNQSIDISKPTRLGTRTQSEADYD
SOV-57      GGIEGWCQKLWTTEAIALLDTVAVKGRFQLTSLINGDNQSIDISKPTRLGTRTQSEADYD
KSOV-2202   GGIEGWCQKLWTTEAIALLDTVAVKGRFQLTSLINGDNQSIDISKPTRLGTRTQSEADYD
```

```
                   790       800       810       820       830       840
PVM-3666    LAINSLRLISAAYKGIGHKLKEGETYLSRDMQFMSKTIQHEGVYYPASIKKILRVGPWIN
PVM-15      LAINSLRLISAAYKGIGHKLKEGETYLSRDMQFMSKTIQHEGVYYPASIKKILRVGPWIN
CPV         LAINSLRLISAAYRGIGHKLKEGETYLSRDMQFMSKTIQHEGVYYPASIKKILRVGPWIN
KSOV-2201   LAINSLRLISAAYKGIGHKLKEGETYLSRDMQFMSKTIQHEGVYYPASIKKILRVGPWIN
SOV-57      LAINSLRLISAAYKGIGHKLKEGETYLSRDMQFMSKTIQHEGVYYPASIKKILRVGPWIN
KSOV-2202   LAINSLRLISAAYKGIGHKLKEGETYLSRDMQFMSKTIQHEGVYYPASIKKILRVGPWIN
```

```
                   850       860       870       880       890       900
PVM-3666    TILDDIKTSTESIGSLTQELEYKGESLMSSLLLRNFWLYRLYSVDLKDHSLCGKQLYRSL
PVM-15      TILDDIKTSTESIGSLTQELEYKGESLMSSLLLRNFWLYRLYSVDLKDHSLCGKQLYRSL
CPV         TILDDIKTSTESIGSLTQELEYKGESLMSSLLLRNFWLYRLYSVDLKDHSLCGKQLYRSL
KSOV-2201   TILDDIKTSTESIGSLTQELEYKGESLMSSLLLRNFWLYRLYSVDLKDHSLCGKQLYRSL
SOV-57      TILDDIKTSTESIGSLTQELEYKGESLMSSLLLRNFWLYRLYSVDLKDHSLCGKQLYRSL
KSOV-2202   TILDDIKTSTESIGSLTQELEYKGESLMSSLLLRNFWLYRLYSVDLKDHSLCGKQLYRSL
```

```
                   910       920       930       940       950       960
PVM-3666    IKVLKHLKRCFNLENLGECLELFLNVPMQFGGADPNVIYRSFYRRTPDFLTESITHLILI
PVM-15      IKVLKHLKRCFNLENLGECLELFLNVPMQFGGADPNVIYRSFYRRTPDFLTESITHLILI
CPV         IKVLKHLKRCFNLENLGECLELFLNVPMQFGGADPNVLYRSFYRRTPDFLTETITHLILI
KSOV-2201   MKVLKHLKRCFNLENLGECLELYLNVPMQFGGADPNVLYRSFYRRTPDFLTESITHLILI
SOV-57      MKVLKHLKRCFNLENLGECLELYLNVPMQFGGADPNVLYRSFYRRTPDFLTESITHLILI
KSOV-2202   MKVLKHLKRCFNLENLGECLELFLNVPMQFGGADPNVLYRSFYRRTPDFLTESITHLILI
```

L

```
                   970       980       990      1000      1010      1020
PVM-3666  LKHFRRDLEFNKDNVSKAVLSLLEFTKNDSAEFVTLMRDPQAIGSERQAKITSDINRTAV
PVM-15    LKHFRRDLEFNKDNVSKAVLSLLEFTKNDSAEFVTLMRDPQAIGSERQAKITSDINRTAV
CPV       LKHFRRDLEFNKDNVSKAVLSLLEFTKNDSAEFVTLMRDPQAIGSERQAKITSDINRTAV
KSOV-2201 LKHFKHDIEFNKDNVSRAVLSLLEFTKNDSAEFVTLMRDPQAIGSERQAKITSDINRTAV
SOV-57    LKHFKQDIEFNKDNVSRAVLSLLEFTKNDSAEFVTLMRDPQAIGSERQAKITSDINRTAV
KSOV-2202 LKHFKRDIEFNKDNVSKAVLSLLEFTKNDSAEFVTLMRDPQAIGSERQAKITSDINRTAV

                  1030      1040      1050      1060      1070      1080
PVM-3666  TSVLSNAPNEIFRTSALHYSSTENELNGIASGVSPVYPHGLRVLYESLPFYKAEKIVNMV
PVM-15    TSVLSNAPNEIFRTSALHYSSTENELNGIASGVSPVYPHGLRVLYESLPFYKAEKIVNMV
CPV       TNVLSNAPNEIFRTSALHYSSTENELNSIASDISPVYPHGLRVLYESLPFHKAEKIVNMV
KSOV-2201 TSVLSNAPNEIFRTSAMHYSTTENELNGIASEINPVYPHGLRVLYESLPFHKAEKIVNMV
SOV-57    TSVLSNAPNEIFRTSAMHYSTTENELNGIASEINPVYPHGLRVLYESLPFHKAEKIVNMV
KSOV-2202 TSVLSNAPNEIFRTSAMHYSTTENELNGIASEINPVYPHGLRVLYESLPFHKAEKIVNMV

                  1090      1100      1110      1120      1130      1140
PVM-3666  SGTKSITNILEKTSAISYTDIIRATNMMVENLTLLTRIMKPGADTSLDPDTIVITILSKI
PVM-15    SGTKSITNILEKTSAISYTDIIRATNMMVENLTLLTRIMKPGADTSLDPDTIVITILSKI
CPV       SGTKSITNILEKTSAISYTDIIRATNMMVENLTLLTRIMKPGADTSLDPDTIVITILSKI
KSOV-2201 SGTKSITNILEKTSAISYTDIIRATNMMVENLTLLARIMKPGADTSLDPDTIVVTILSKI
SOV-57    SGTKSITNILEKTSAISYTDIIRATNMMVENLTLLARIMKPGADTSLDPDTIVVTILSKI
KSOV-2202 SGTKSITNILEKTSAISYTDIIRATNMMVENLTLLTRIMKPGADTSLDPDTIVVTILSKI

                  1150      1160      1170      1180      1190      1200
PVM-3666  IRDKSWDVGDIIGVTSPSPVSCFKVVYTSTLQNNSVVIERYTTDTYTRGKRGPTKPWVGS
PVM-15    IRDKSWDVGDIIGVTSPSPVSCFKVVYTSTLQNNSVVIERYTTDTYTRGKRGPTKPWVGS
CPV       IRDKSWDVGDIIGVTSPSPVSCFKVVYTSTLQNNSVVIERYTTDTYTRGKRGPTKPWVGS
KSOV-2201 IRDKSWDVGDIIGVTSPSPVSCFKVVYTSTLQNNSVVIERYTADTYTRGKRGPTKPWVGS
SOV-57    IRDKSWNVGDIIGVTSPSPVSCFKVVYTSTLQNNSVVIERYTTDTYTRGKRGPTKPWVGS
KSOV-2202 IRDKSWDVGDIIGVTSPSPVSCFKVVYTSTLQDNSVVIERYTTDTYTRGKRGPTKPWVGS

                  1210      1220      1230      1240      1250      1260
PVM-3666  STQEKKSMPVYNRQVLTRGQRDQIENIAKLEWVFSSVANIDSLLNELSTMTLGLSLRKCR
PVM-15    STQEKKSMPVYNRQVLTRGQRDQIENIAKLEWVFSSVANIDSLLNELSTMTLGLSLRKCR
CPV       STQEKKSMPVYNRQVLTRGQRDQIENIAKLEWVFSSVANIDSLLNELSTMTLGLSLRKCK
KSOV-2201 STQEKKSMPVYNRQVLTRSQRDQIENIAKLDWVFSSVVNIDPLLNELSTMTLGLSLRKCR
SOV-57    STQEKKSMPVYNRQVLTRGQRDQIENIAKLDWVFSSVVNIDPLLNELSTMTLGLSLRKCR
KSOV-2202 STQEKKSMPVYNRQVLTRGQRDQIENIAKLDWVFGSVINIDPLLDELSTMTLGLSLKKCR

                  1270      1280      1290      1300      1310      1320
PVM-3666  QLFPTYLSLNFLHRLSVSSRPREYPSSLPAYRTTNFHFDTGPINKVLTERFGDEDINLVF
PVM-15    QLFPTYLSLNFLHRLSVSSRPREYPSSLPAYRTTNFHFDTGPINKVLTERFGDEDINLVF
CPV       QLFPTYLSLNFLHRLSVSSRPREYPSSLPAYRTTNFHFDTGPINKVLTERFGDEDINLVF
KSOV-2201 QLFPTYLSLNFLHRLSVSSRPREYPSSLPAYRTTNFHFDTSPINKVLTERFGDEDINLVF
SOV-57    QLFPTYLSLNFLHRLSVSSRPREYPSSLPAYRTTNFHFDTSPINKVLTERFGDEDINLVF
KSOV-2202 QLFPTYLSLNFLHRLSVSSRPREYPSSLPAYRTTNFHFDTGPINKVLTERFGDEDINLVF

                  1330      1340      1350      1360      1370      1380
PVM-3666  QNAISYGLSTMSLVEQFTGVCPNKVLLVPKLQEIQLMKVPIFQGGFNLQSIIPIIRQQHM
PVM-15    QNAISYGLSTMSLVEQFTGVCPNKVLLVPKLQEIQLMKVPIFQGGFNLQSIIPIIRQQHM
CPV       QNAISYGLSTMSLVEQFTGVCPNKVLLVPKLQEIQLMKVPIFQGGFNLQSIIPIIRQQHM
KSOV-2201 QNAISYGLSTMSLVEQFTGVCPNKVLLVPKLQEIQLMKVPIFQGEFNLQSIIPIIRQQHM
SOV-57    QNAISYGLSTMSLVEQFTGVCPNKVLLVPKLQEIQLMKVPIFQGEFNLQSIIPIIRQQHM
KSOV-2202 QNAISYGLSTMSLVEQFTGVCPNKVLLVPKLQEIQLMKVPIFQGEFNLQSIIPIVRQQHM

                  1390      1400      1410      1420      1430      1440
PVM-3666  FLPNHITPAQYIELFLSSKQFHSRINLKHNNRFKLVLQKDYFNGENMIETLSTCLAGHWI
PVM-15    FLPNHITPAQYIELFLSSKQFHSRINLKHNNRFKLVLQKDYFNGENMIETLSTCLAGHWI
CPV       FLPNHITPAQYIELFLSSKQFHTRINLNHNNRFKLVLQKDYFNGENMIETLSTCLAGHWI
KSOV-2201 FLPNHITPAQYIELFLSSKQFHSQINLKYNNRFKLVLQKDYFNGENMVETLSTCLAGHWI
SOV-57    FLPNHITPAQYIELFLSSKQFHSQINLKYNNRFKLVLQKDYFNGENMVETLSTCLAGHWI
KSOV-2202 FLPNHITPAQYIELFLSSKQFHSQINLKHNNRFKLVLQKDYFNGENMVETLSTCLAGHWI
```

L

```
                 1930      1940      1950      1960      1970      1980
PVM-3666    SGINTNIKSLIPMLDYPITYNKITTLLESVRELSSNKNKNTMWIGRNPVYHNKWLKRKYF
PVM-15      SGINTNIKSLLPMLDYPITYNKITTLLESVRELSSNKNKNTMWIGRNPVYHNKWLKRKYF
CPV         SGINTNIKSLIPMLDYPITYNKITTLLESVRELSSNKNKNTMWIGRNPVYHNKWLKRKYF
KSOV-2201   SGINTNIKSLIPMLDYPITYNKITTLLESVRELSPNKN..TMWIGRNPVYHNKWLRRKYF
SOV-57      SGINTNIKSLIPMLDYPITYNKITTLLESVRELSPNKN..TMWIGRNPVYHNKWLRRKYF
KSOV-2202   SGINTNIKSLIPMLDYPITYNKITTLLESVRELSPNKN..TMWIGRNPVYHNKWLKRKYF

                 1990      2000      2010      2020      2030      2040
PVM-3666    NILKWLKYCIELPAFRMDYNSFERIEMLYPNLRDLVDSVSTSELKKVIKVTGILFRSNTM
PVM-15      NILKWLKYCIELPAFRMDYNSFERIEMLYPNLRDLVDSVSTSELKKVIKVTGILFRSNTM
CPV         NILKWLKYCIELPAFRMDYNSFERIEMLYPNLRDLVDSVSTSELKKVVKVTGILFRSNTM
KSOV-2201   NILKWLKYCIELPAFRMDYNSFERIEMLYPNLRDLVDSVSTSELKKVIKVTGVLFRSDTM
SOV-57      NILKWLKYCIELPAFRMDYNSFERIEMLYPNLRDLVDSVSTSELKKVIKVTGVLFRSDTM
KSOV-2202   NILKWLKYCIELPAFRMDYNSFERIEMLYPNLRDLVDSVSTSELKKVIKVTGILFRSDTM
```

P

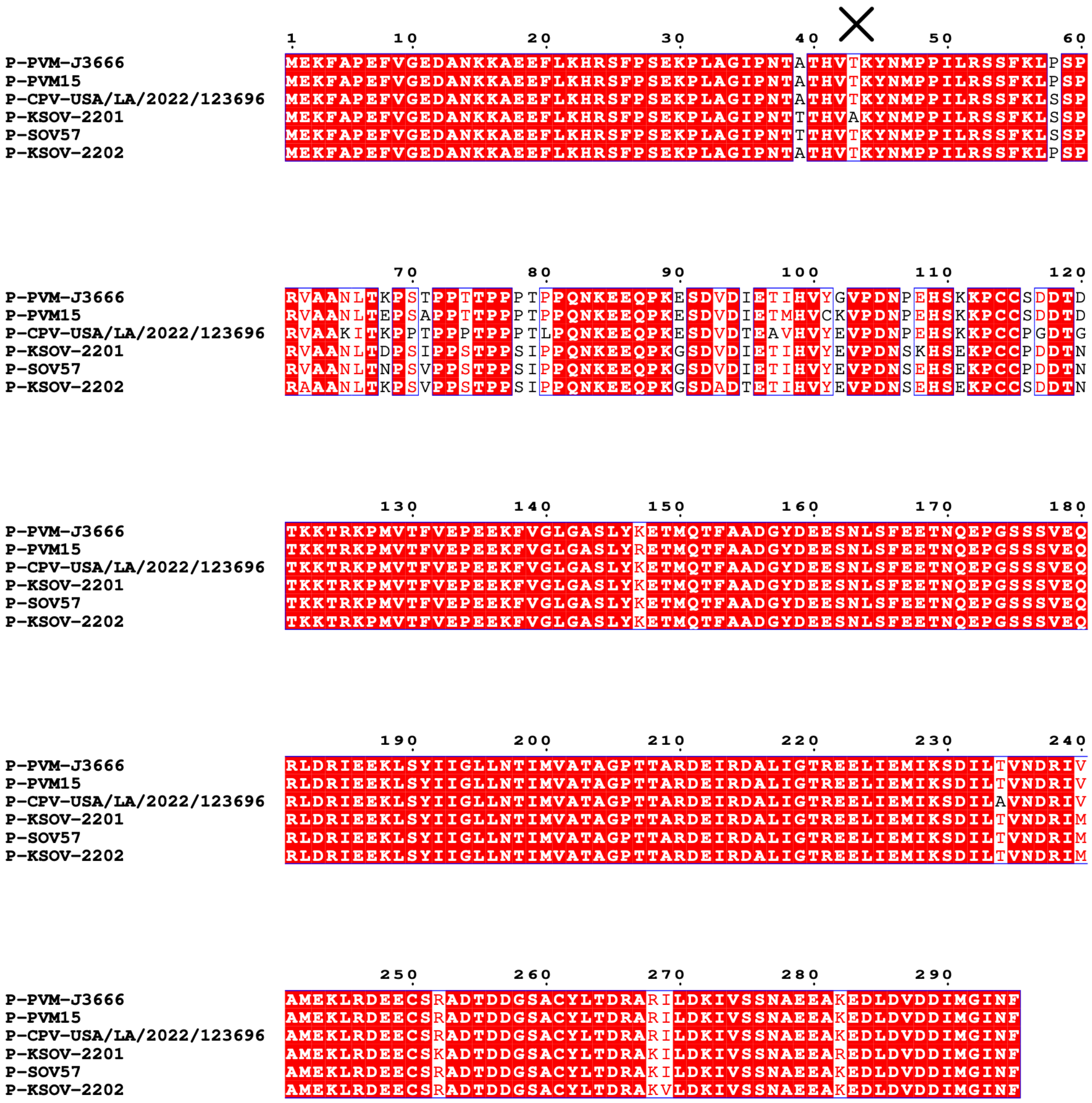

1 10 20 30 40 50 60
P-PVM-J3666 MEKFAPEFVGEDANKKAEEFLKHRSFPSEKPLAGIPNTATHVTKYNMPPILRSSFKLPSP
P-PVM15 MEKFAPEFVGEDANKKAEEFLKHRSFPSEKPLAGIPNTATHVTKYNMPPILRSSFKLPSP
P-CPV-USA/LA/2022/123696 MEKFAPEFVGEDANKKAEEFLKHRSFPSEKPLAGIPNTATHVTKYNMPPILRSSFKLSSP
P-KSOV-2201 MEKFAPEFVGEDANKKAEEFLKHRSFPSEKPLAGIPNTTTHVAKYNMPPILRSSFKLSSP
P-SOV57 MEKFAPEFVGEDANKKAEEFLKHRSFPSEKPLAGIPNTTTHVTKYNMPPILRSSFKLSSP
P-KSOV-2202 MEKFAPEFVGEDANKKAEEFLKHRSFPSEKPLAGIPNTATHVTKYNMPPILRSSFKLPSP
70 80 90 100 110 120
P-PVM-J3666 RVAANLTKPSTPPTTPPPTPPQNKEEQPKESDVDIETIHVYGVPDNPEHSKKPCCSDDTD
P-PVM15 RVAANLTEPSAPPTTPPPTPPQNKEEQPKESDVDIETMHVCKVPDNPEHSKKPCCSDDTD
P-CPV-USA/LA/2022/123696 RVAAKITKPPTPPPTPPPTLPQNKEEQPKESDVDTEAVHVYEVPDNPEHSKKPCCPGDTG
P-KSOV-2201 RVAANLTDPSIPPSTPPSIPPQNKEEQPKGSDVDIETIHVYEVPDNSKHSEKPCCPDDTN
P-SOV57 RVAANLTNPSVPPSTPPSIPPQNKEEQPKGSDVDIETIHVYEVPDNSEHSEKPCCPDDTN
P-KSOV-2202 RAAANLTKPSVPPSTPPSIPPQNKEEQPKGSDADTETIHVYEVPDNSEHSEKPCCSDDTN
130 140 150 160 170 180
P-PVM-J3666 TKKTRKPMVTFVEPEEKFVGLGASLYKETMQTFAADGYDEESNLSFEETNQEPGSSSVEQ
P-PVM15 TKKTRKPMVTFVEPEEKFVGLGASLYRETMQTFAADGYDEESNLSFEETNQEPGSSSVEQ
P-CPV-USA/LA/2022/123696 TKKTRKPMVTFVEPEEKFVGLGASLYKETMQTFAADGYDEESNLSFEETNQEPGSSSVEQ
P-KSOV-2201 TKKTRKPMVTFVEPEEKFVGLGASLYKETMQTFAADGYDEESNLSFEETNQEPGSSSVEQ
P-SOV57 TKKTRKPMVTFVEPEEKFVGLGASLYKETMQTFAADGYDEESNLSFEETNQEPGSSSVEQ
P-KSOV-2202 TKKTRKPMVTFVEPEEKFVGLGASLYKETMQTFAADGYDEESNLSFEETNQEPGSSSVEQ
190 200 210 220 230 240
P-PVM-J3666 RLDRIEEKLSYIIGLLNTIMVATAGPTTARDEIRDALIGTREELIEMIKSDILTVNDRIV
P-PVM15 RLDRIEEKLSYIIGLLNTIMVATAGPTTARDEIRDALIGTREELIEMIKSDILTVNDRIV
P-CPV-USA/LA/2022/123696 RLDRIEEKLSYIIGLLNTIMVATAGPTTARDEIRDALIGTREELIEMIKSDILAVNDRIV
P-KSOV-2201 RLDRIEEKLSYIIGLLNTIMVATAGPTTARDEIRDALIGTREELIEMIKSDILTVNDRIM
P-SOV57 RLDRIEEKLSYIIGLLNTIMVATAGPTTARDEIRDALIGTREELIEMIKSDILTVNDRIM
P-KSOV-2202 RLDRIEEKLSYIIGLLNTIMVATAGPTTARDEIRDALIGTREELIEMIKSDILTVNDRIM
250 260 270 280 290
P-PVM-J3666 AMEKLRDEECSRADTDDGSACYLTDRARILDKIVSSNAEEAKEDLDVDDIMGINF
P-PVM15 AMEKLRDEECSRADTDDGSACYLTDRARILDKIVSSNAEEAKEDLDVDDIMGINF
P-CPV-USA/LA/2022/123696 AMEKLRDEECSRADTDDGSACYLTDRARILDKIVSSNAEEAKEDLDVDDIMGINF
P-KSOV-2201 AMEKLRDEECSKADTDDGSACYLTDRAKILDKIVSSNAEEAREDLDVDDIMGINF
P-SOV57 AMEKLRDEECSRADTDDGSACYLTDRAKILDKIVSSNAEEAKEDLDVDDIMGINF
P-KSOV-2202 AMEKLRDEECSRADTDDGSACYLTDRAKVLDKIVSSNAEEAKEDLDVDDIMGINF

N

```
               1        10        20        30        40        50        60
PVM-15-N       MSLDRLKLNDVSNKDSLLSNCKYSVTRSTGDVTSVSGHAMQKALARTLGMFLLTAFNRCE
PVM-J3666-N    MSLDRLKLNDVSNKDSLLSNCKYSVTRSTGDVTSVSGHAMQKALARTLGMFLLTAFNRCE
KSOV-2201-N    MSLDRLKLNDVSNKDSLLSNCKYSVTRSTGDVTSVSGHAMQKALARTLGMFLLTAFNRCE
KSOV57-N       MSLDRLKLNDVSNKDSLLSNCKYSVTRSTGDVTSVSGHAMQKALARTLGMFLLTAFNRCE
KSOV-2202-N    MSLDRLKLNDVSNKDSLLSNCKYSVTRSTGDVTSVSGHAMQKALARTLGMFLLTAFNRCE
CPV-N          MSLDKLKLNDVSNKDSLLSNCKYSVTRSTGDVTSVSGHAMQKALARTLGMFLLTAFNRCE
```

```
                        70        80        90       100       110       120
PVM-15-N       EVAEIGLQYAMSLLGRDDSIKILREAGYNVKCVDTQLKDFTIKLQGKEYKIQVLDIVGID
PVM-J3666-N    EVAEIGLQYAMSLLGRDDSIKILREAGYNVKCVDTQLKDFTIKLQGKEYKIQVLDIVGID
KSOV-2201-N    EVAEIGLQYAMSLLGRDDSIKILREAGYNVKCVDTQLKDFTIKLQGKEYKIQVLDIVGID
KSOV57-N       EVAEIGLQYAMSLLGRDDSIKILREAGYNVKCVDTQLKDFTIKWQGKEYKIQVLDIVGID
KSOV-2202-N    EVAEIGLQYAMSLLGRDDSIKILREAGYNVKCVDTQLKDFTIKLQGKEYKIQVLDIVGVD
CPV-N          EVAEIGLQYAMSLLGRDDSIKILREAGYNVKCVDTQLKDFTIKLQGKDYKIQVLDIVGID
```

```
                       130       140       150       160       170       180
PVM-15-N       AANLADLEIQARGVVAKELKTGARLPDNRRHDAPDCGVIVLCIAALVVSKLAAGDRGGLD
PVM-J3666-N    AANLADLEIQARGVVAKELKTGARLPDNQRHDAPDCGVIVLCIAALVVSKLAAGDRGGLD
KSOV-2201-N    AANLAELEIQARGVVAKELKTGAGLPDNRRHDAPDCGVIVLCVAALVVSKLAAGDRGGLD
KSOV57-N       AANLAELEIQARGVVAKELKTGAGLPDNRRHDAPDCGVIVLCVAALVVSKLAAGDRGGLD
KSOV-2202-N    AANLANLEIQARGVVAKELKTGAGLPDNRRHDAPDCGVIVLCVAALVVSKLAAGDRGGLD
CPV-N          AANLADLEIQARGVVAKELKTGAGLPDNRRHDAPDYGVIVVCGAALVVSKLAAGDRGGLD
```

```
                       190       200       210       220       230       240
PVM-15-N       AVERRALNVLKAEKARYPNMEVKQIAESFYDLFERKPYYIDVFITFGLAQSSVKGGSKVE
PVM-J3666-N    AVERRALNVLKAEKARYPNMEVKQIAESFYDLFERKPYYIDVFITFGLAQSSVKGGSKVE
KSOV-2201-N    AVERRALNVLKAEKARYPNLEVKQIAESFYDLFERKPYYIDVFITFGLAQSSVKGGSKVE
KSOV57-N       AVERRALNVLKAEKARYPNLEVKQIAESFYDLFERKPYYIDVFITFGLAQSSVKGGSKVE
KSOV-2202-N    AVERRALNVLKAEKARYPNLEVKQIAESFYDLFERKPYYIDVFITFGLAQSSVKGGSKVE
CPV-N          AVERRALNVLKAEKARYPNLEVKQIAESFYDLFERKPYYIDIFITFGLAQSSVKGGSKVE
```

```
                       250       260       270       280       290       300
PVM-15-N       GLFSGLFMNAYGAGQVMLRWGLLAKSVKNIMLGHASVQAEMEQVVEVYEYAQKQGGEAGF
PVM-J3666-N    GLFSGLFMNAYGAGQVMLRWGLLAKSVKNIMLGHASVQAEMEQVVEVYEYAQKQGGEAGF
KSOV-2201-N    GLFSGLFMNAYGAGQVMLRWGILAKSVKNIMLGHASVQAEMEQVVEVYEYAQKQGGEAGF
KSOV57-N       GLFSGLFMNAYGAGQVMLRWGLLAKSVKNIMLGHASVQAEMEQVVEVYEYAQKQGGEAGF
KSOV-2202-N    GLFSGLFMNAYGAGQVMLRWGLLAKSVKNIMLGHASVQAEMEQVVEVYEYAQKQGGEAGF
CPV-N          GLFSGLFMNAYGAGQVMLRWGLLAKSVKNIMLGHASVQAEMEQVVEVYEYAQKQGGEAGF
```

```
                       310       320       330       340       350       360
PVM-15-N       YHIRNNPKASLLSLTNCPNFTSVVLGNAAGLGIIGSYKGAPRNRELFDAAKDYAERLKDN
PVM-J3666-N    YHIRNNPKASLLSLTNCPNFTSVVLGNAAGLGIIGSYKGAPRNRELFDAAKDYAERLKDN
KSOV-2201-N    YHIRNNPKASLLSLTNCPNFTSVVLGNAAGLGIIGSYKGAPRNRELFDAAKDYAERLKDN
KSOV57-N       YHIRNNPKASLLSLTNCPNFTSVVLGNAAGLGIIGSYKGAPRNRELFDAAKDYAERLKDN
KSOV-2202-N    YHIRNNPKASLLSLTNCPNFTSVVLGNAAGLGIIGSYKGAPRNRELFDAAKDYAERLKDN
CPV-N          YHIRNNPKASLLSLTNCPNFTSVVLGNAAGLGIIGSYKGAPRNRELFDAAKDYAERLKDN
```

```
                       370       380       390
PVM-15-N       NVINYSALNLTAEERELISQQLNIVDDTPDDDI
PVM-J3666-N    NVINYSALNLTAEERELISQQLNIVDDTPDDDI
KSOV-2201-N    NVINYSALNLTAEERELISQQLDTVDDTPDDDI
KSOV57-N       NVINYSALNLTAEERELISQQLNTVDDTPDDDI
KSOV-2202-N    NVINYSALNLTAEERELISQQLNIVEDTPDDDI
CPV-N          NVINYSALNLTTEERELISQQLNIVDDTPDDDI
```

M2-1

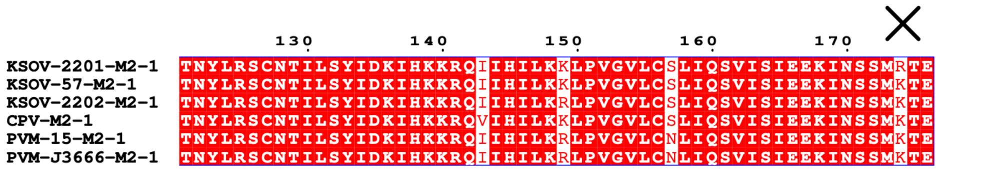

SUPPLEMENTARY FIG 2 Sequence alignments between L, P, N and M2-1 of the three published SOV strains, PVM and CPV sequences. The crosses indicate amino acid residues that diverge for KSOV-2201 from other viruses.

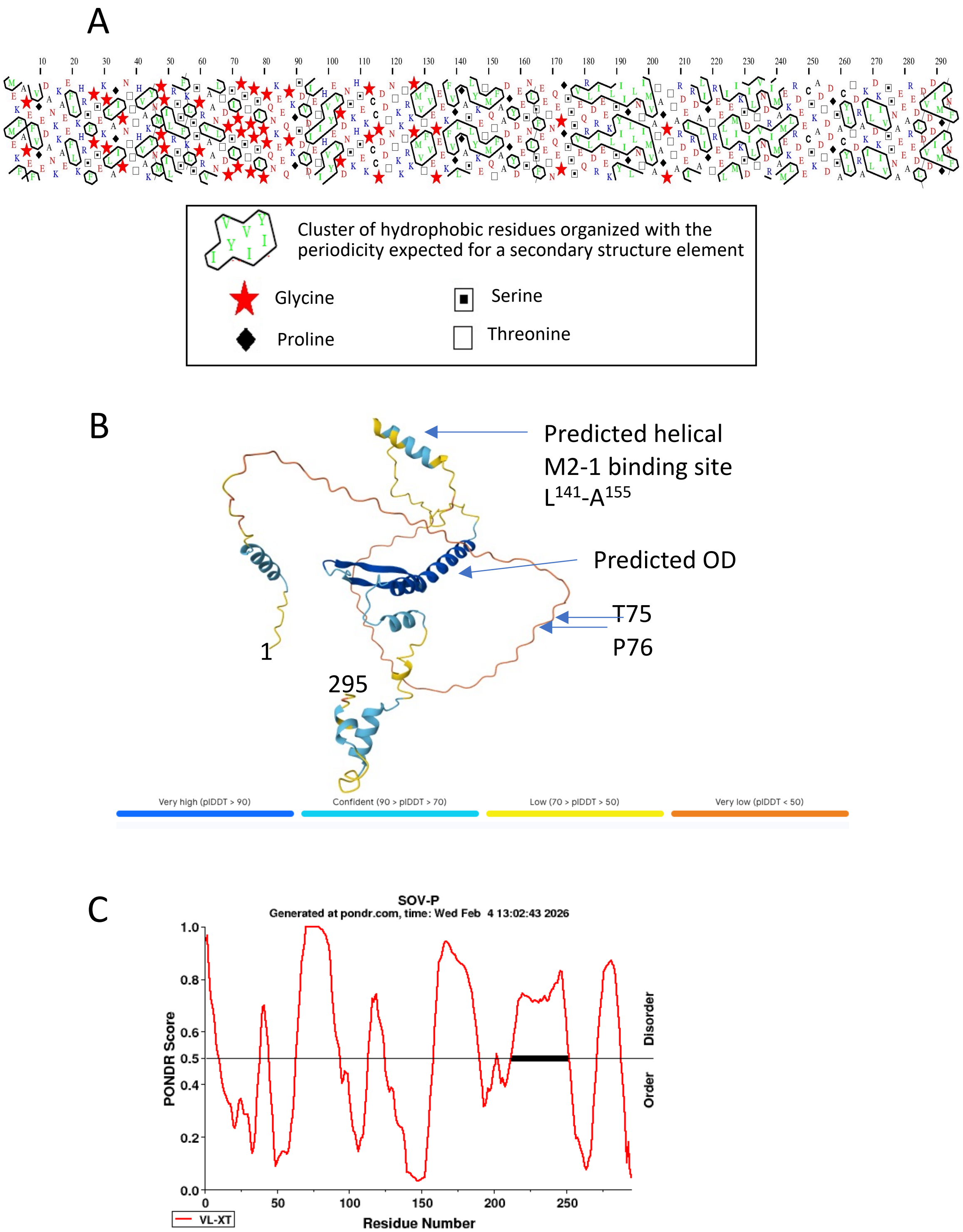

SUPPLEMENTARY FIG 3 Searching for a poorly conserved and disordered region in the SOV P protein for BFP insertion. (A) Hydrophobic cluster analysis plot of the SOV (strain KSOV-2201) P protein sequence. Structured regions are characterized by a high number of hydrophobic clusters (green), whereas unstructured regions have low numbers or are devoid of hydrophobic clusters and are generally rich in proline and glycine residues. **(B)** Structure prediction of SOV P using AlphaFold3 with standard pLDDT coloring, from the Mol* molecular viewer at the AlphaFold server. The protein is colored according to the pLDDT. OD, oligomerization domain. The residues T75 and P76, between which the BFP was inserted, are pointed by arrows. The predicted TM (pTM) score, a single-value metric reflecting the accuracy of the overall predicted structure, is 0.3. **(C)** Prediction of disordered domains for SOV P using the PONDR server. Note that the PONDR score for residues 75-76 is very high (1.0).

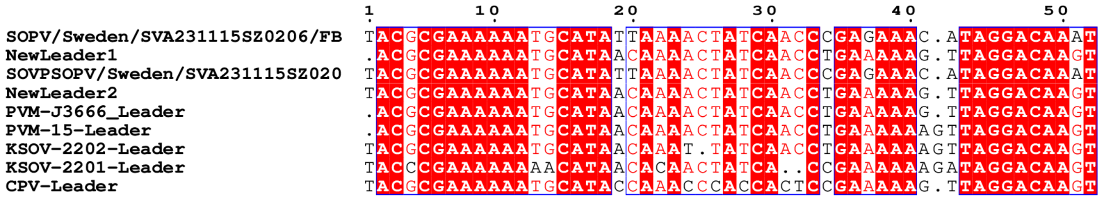

SUPPLEMENTARY FIG 4 Sequence alignments between the published SOV Leader cDNA sequences including the two recently published Swedish sequences and those of one CPV (strain Bari/100-12/ITA/2012), two PVM strains, and the two SOV Leader sequences used for minigenome development. Note the presence of an additional T at the 5' end for the two Swedish strains, the two Korean strains and the CPV strain in the alignment.

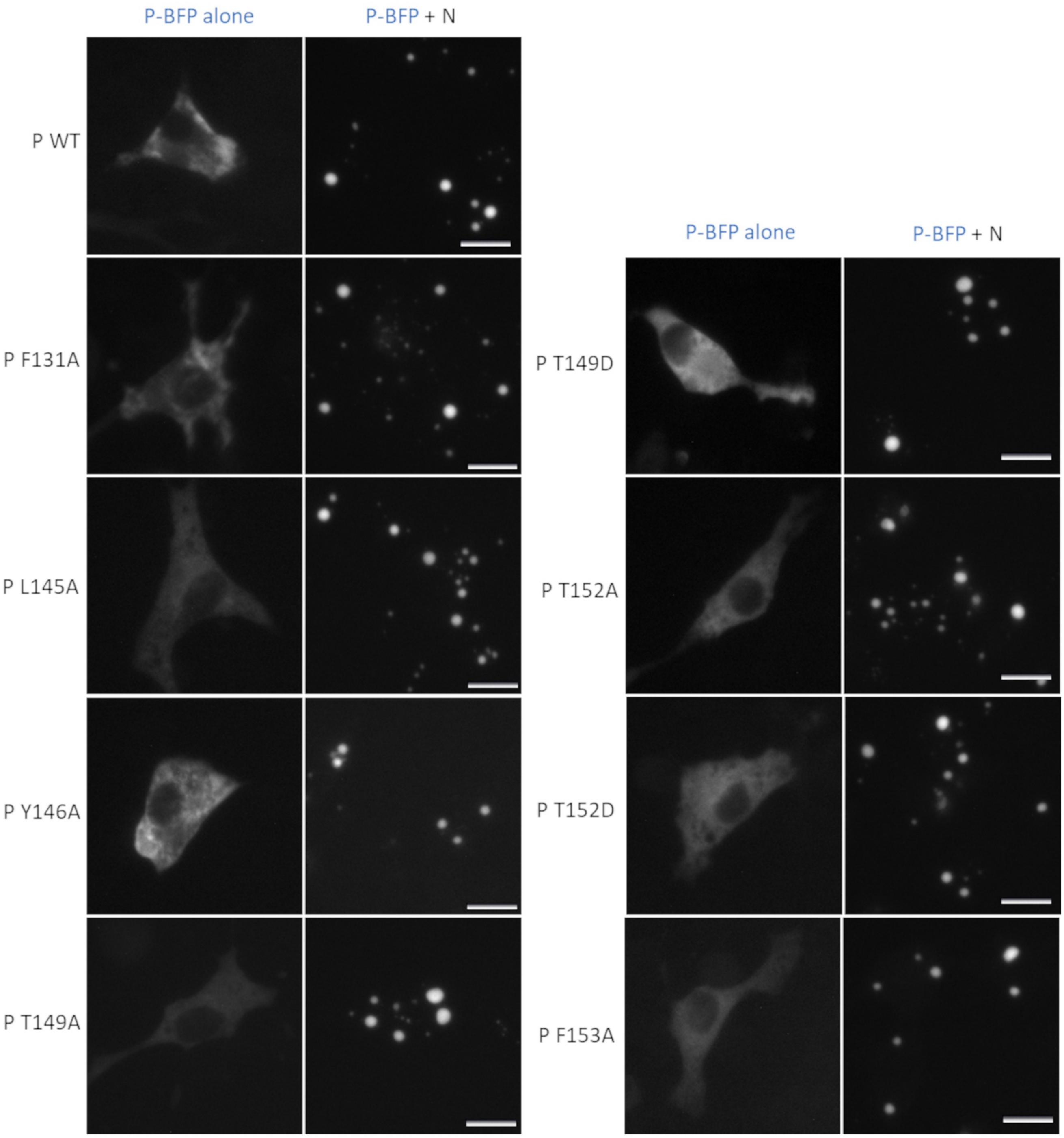

SUPPLEMENTARY FIG 5 The different SOV P-BFP variants mutated at the M2-1- and PP1-binding sites still form IBs but only in the presence of N. BSRT7 cells were transfected with plasmids encoding the N, P-BFP (WT and variants). 24 H post-transfection, cells were fixed and labelled with a rabbit polyclonal anti-N antibody and detected with a secondary goat anti-rabbit antibody. The colocalization of proteins in IBs was analyzed by fluorescence microscopy. Scale bars, 20 µm.

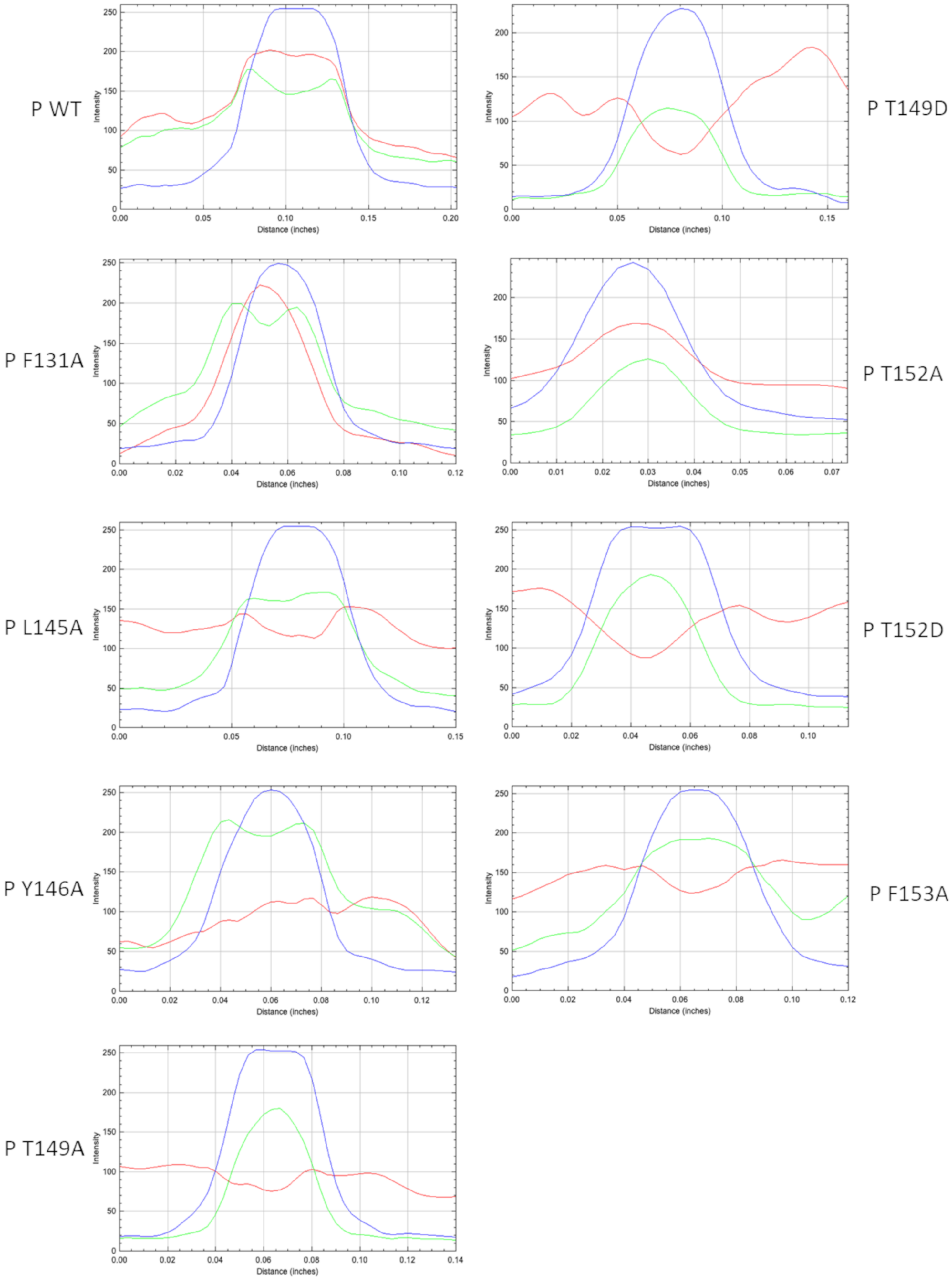

SUPPLEMENTARY FIG 6 Quantification of the presence of M2-1 in IBs depending of P variants.
Red, green and blue fluorescence, corresponding to M2-1, N and P, respectively, was quantified using ImageJ software from pictures shown in Fig. 8 (white bars). The presence of M2-1 in IBs is revealed by a red peak that merges with the blue and green ones.
